# Protein phosphatase 2A regulates cytotoxicity and drug resistance by dephosphorylating AHR and MDR1

**DOI:** 10.1016/j.jbc.2022.101918

**Published:** 2022-04-08

**Authors:** Liping Chen, Ping Guo, Wenxue Li, Xinhang Jiang, Qun Zhao, Daochuan Li, Qing Wang, Yongmei Xiao, Xiumei Xing, Yaqin Pang, Michael Aschner, Lihua Zhang, Wen Chen

**Affiliations:** 1 Guangdong Provincial Key Laboratory of Food, Nutrition and Health, Department of Toxicology, School of Public Health, Sun Yat-sen University, Guangzhou, China; 2 Department of Toxicology, Guangzhou Center for Disease Control and Prevention, Guangzhou, China; 3 Key Laboratory of Separation Science for Analytical Chemistry, Dalian Institute of Chemical Physics, Chinese Academy of Science, National Chromatographic Research and Analysis Center, Dalian, China; 4 Faculty of Toxicology, School of Public Health, Youjiang Medical College for Nationalities, Guangxi, China; 5 Department of Molecular Pharmacology, Albert Einstein College of Medicine, Bronx, New York, USA

**Keywords:** protein phosphatase 2A, metabolic enzymes, xenobiotic metabolism, drug resistance, regulatory mode, hepatotoxicity, ADR, adriamycin, AHR, aryl hydrocarbon receptor, BaP, benzo(a)pyrene, BMD, benchmark dose, CAR, constitutive active androstane receptor, co-IP, coimmunoprecipitation, CYP, cytochrome P450, DEG, differentially expressed gene, DEPP, differentially expressed phosphoprotein, HNF-4α, hepatocyte nuclear factor 4-alpha, HO, homozygous knockout, IPA, Ingenuity Pathway Analysis, KEGG, Kyoto Encyclopedia of Genes and Genomes, MMP, mitochondrial membrane potential, MS/MS, tandem mass spectrometry, p-AHR, phosphorylated AHR, PP2A, protein phosphatase 2A, PTM, post-translational modification, Rh123, rhodamine 123, ROS, reactive oxygen species

## Abstract

Protein phosphatase 2A (PP2A) is a serine/threonine dephosphorylating enzyme complex that plays numerous roles in biological processes, including cell growth and metabolism. However, its specific actions in many of these critical pathways are unclear. To explore mechanisms underlying metabolic enzyme regulation in the liver, we investigated the key pathways involved in regulation of xenobiotic-metabolizing enzymes in a mouse model with hepatocyte-specific deletion of *Ppp2r1a*, encoding the Aα subunit of PP2A. We performed transcriptome and phosphoproteome analysis in mouse livers at the age of 3 months and identified 2695 differentially expressed genes and 549 upregulated phosphoproteins in homozygous knockout mouse livers compared with WT littermates. In particular, the expression of metabolic enzymes Cyp2e1, Cyp1a1, Cyp1a2, Mdr1a, and Abcg2 was dramatically altered in homozygous knockout mouse livers. We also demonstrated that activation of PP2A reversed the decline of metabolic enzyme expression in primary mouse hepatocytes. We found that specific PP2A holoenzymes were involved in metabolic enzyme induction through dephosphorylation of transcription factors, nuclear receptors, or the target enzymes themselves, leading to dysregulation of xenobiotic metabolism or drug-induced hepatotoxicity. Notably, we confirmed that a regulatory axis, PP2A B56α–aryl hydrocarbon receptor–Cyp1a1, was involved in benzo(a)pyrene-induced cytotoxicity through dephosphorylation of the metabolic nuclear receptor, aryl hydrocarbon receptor, at serine 36. In addition, we showed that PP2A B56δ complexes directly dephosphorylated the multidrug efflux pump MDR1 (encoded by multi-drug resistance gene 1), contributing to drug resistance against the chemotherapeutic 5-fluorouracil. Taken together, these novel findings demonstrate the involvement of PP2A in the regulation of liver metabolism.

Biotransformation is essential in mediating biological effects in response to xenobiotics, environmental pollutants, or drugs, and it depends on the activity of metabolic enzymes (1). The metabolism or transport of xenobiotics is critical for drug clearance, pharmacokinetics, bioavailability, target organ toxicity, and/or therapeutic efficacy (2, 3). The majority of metabolism is catalyzed by enzymes belonging to the cytochrome P450 (CYP) superfamily, which have been well characterized (4). Exposure to xenobiotics leads to altered gene expression profile of metabolic enzymes, thereby regulating the activation, detoxification, or transport of xenobiotic, conferring cells with adaptive responses to potential damages. Dysregulation of metabolic enzymes has been linked to many human diseases, such as cancer, diabetes, and nonalcoholic fatty liver disease (5, 6). However, the mechanism associated with regulation of metabolic enzymes in response to environmental pollutants or pharmaceuticals is not completely understood.

Exposure to environmental factors or drugs leads to alteration in activity of metabolic enzymes through regulation in nuclear receptors, transcription factors, epigenetic modifications, and protein stabilization (7, 8). For instance, metabolic nuclear receptors, including constitutive active androstane receptor (CAR), pregnant X receptor, aryl hydrocarbon receptor (AHR), and peroxisome proliferator–activated receptors (s) have been implicated in regulation of metabolic enzyme activity following exposure to xenobiotics (9, 10). The nuclear receptors function as master regulators of a metabolizing network, leading to alterations in drug resistance, energy metabolism, and lipid homeostasis *via* binding of their cognate ligands (11, 12). In addition, the metabolic enzymes have been shown to be transcriptionally induced by several liver-specific transcription factors, such as CCAAT/enhancer-binding protein beta and hepatocyte nuclear factor 4-alpha (HNF-4α) (13). Moreover, trichostatin A, a histone deacetylase inhibitor, has been shown to preserve the differentiated hepatic phenotype through regulation of the expression of these transcription factors (14). Metabolic enzymes involved in endobiotic and xenobiotic metabolism are transcriptionally regulated, but other processes have been implicated in as well. For example, it has been shown that numerous metabolic enzymes are specifically ubiquitinated by an E3 ubiquitin ligase and targeted for proteasomal degradation for the acclimation of cells in response environmental stimuli (15, 16). Moreover, accumulating evidence suggests that post-translational modifications (PTMs) are involved in regulation of metabolic reprogramming (17). However, the mode of action of PTMs underlying xenobiotic-induced metabolic regulation has yet to be characterized.

The accumulated evidence indicates that xenobiotic metabolism may be regulated through activation or inhibition of signaling pathways, such as PKC, p38 mitogen-activated protein kinase, glycogen synthase kinase-3β, or C-Jun N-terminal kinases, where phosphorylation of these kinases are involved in mediating the transcriptional activation of specific metabolic enzymes (18, 19, 20, 21). Thus, the phosphorylation modifications play an important role in regulation of metabolic enzymes. Protein phosphatase 2A (PP2A) is a major serine/threonine phosphatase family in eukaryotic cells with a heterotrimeric holoenzyme comprised of a scaffolding A subunit, catalytic C subunit, and one of several regulatory B subunits (22). Distinct PP2A holoenzymes are implicated in a series of cellular events, including cell cycle, DNA replication, cell growth, and apoptosis (23, 24). Perturbation of signaling pathways governed by specific PP2A complexes has been linked to the development and progression of human diseases (23, 25). Prior study has revealed that PP2A-mediated dephosphorylation of Sp1 at serine 59 was critical in tetrachlorodibenzo-p-dioxin-induced Cyp1a1 transcription (26). Our prior work also revealed that PP2A-mediated dephosphorylation of β-catenin was involved in regulation of benzene-induced hematotoxicity through suppressing Cyp2e1 expression (27). In this regard, we speculated that PP2A might be a key modulator in regulation of the xenobiotic metabolism. To date, the key signaling pathways governed by specific PP2A holoenzymes have yet to be defined. Previously, we developed a mouse model with hepatocyte-specific deletion in *Ppp2r1a* gene (encoding PP2A Aα subunit). Using this model, we identify the critical pathways regulated by specific PP2A holoenzymes in development of liver fibrosis (28). Here, we integrated the datasets from transcriptome and phosphoproteome to profile the metabolic enzymes in this mouse model and characterized the regulatory role of PP2A in xenobiotic metabolism and related biological consequences. Notably, we showed that two specific regulatory modes participated in regulation of xenobiotic-induced activation of metabolic enzymes, leading to alterations in chemical-induced cytotoxicity and drug resistance. The understanding of PP2A-mediated signaling pathways highlights the key phosphatases and pathways involved in regulation of xenobiotic metabolism or drug resistance.

## Results

### Identification of PP2A-regulated metabolic signaling pathways

**Figure 1:**
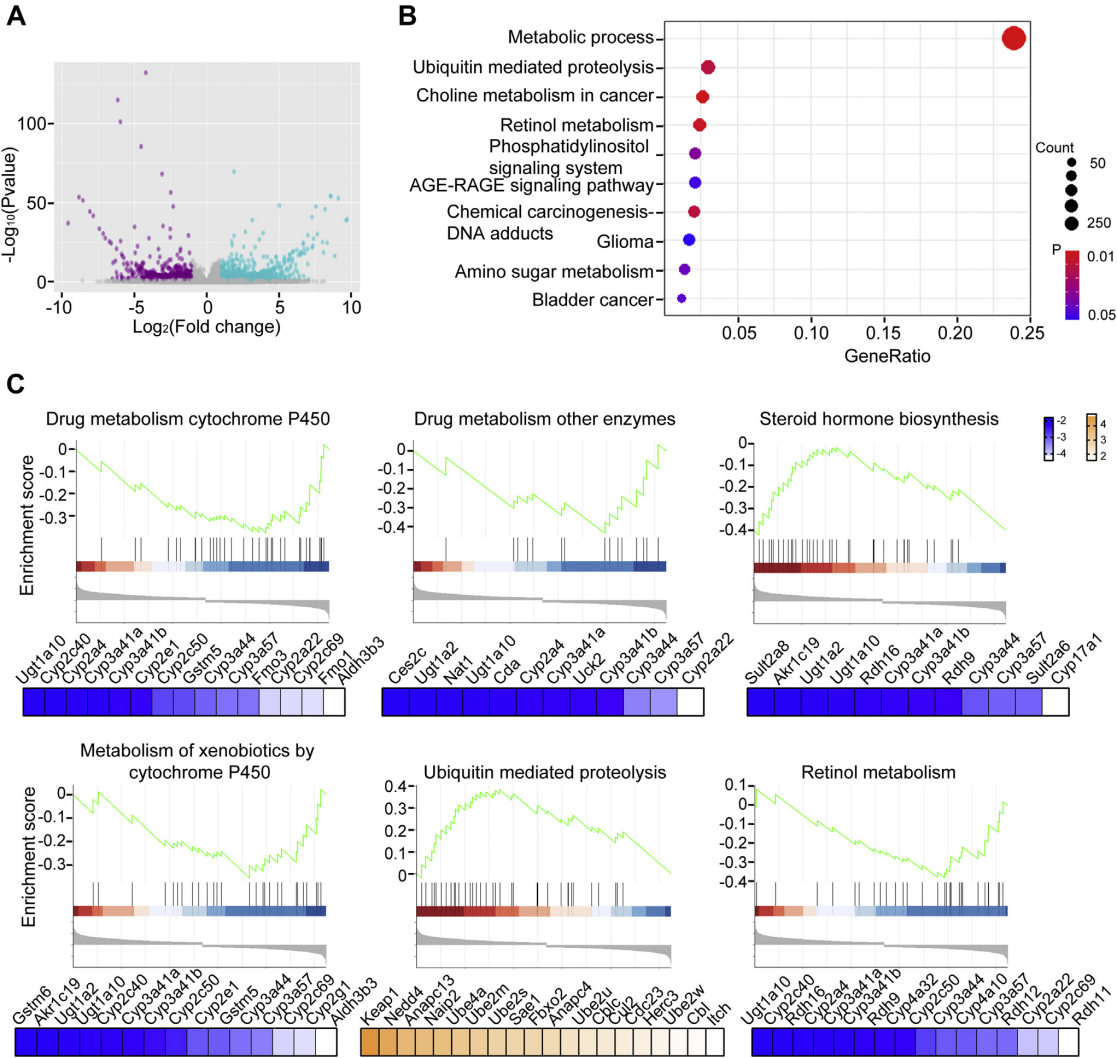
**PP2A was involved in regulation of hepatic metabolism.***A*, volcano plots. Significantly upregulated and downregulated genes in liver tissues from HO mouse compared with that in WT mouse are shown in *purple* and *blue*, respectively. The thresholds were a |fold change| >2 and *p* < 0.05. *B*, scatter plot of top 10 enriched KEGG pathways for differentially expressed genes (DEGs). The size of the *circles* represents the numbers of enriched genes in analysis. *C*, gene set enrichment analysis (GSEA)-enrichment plots of representative gene sets for DEGs. Significant changes in gene expression of each gene set were indicated as upregulated (*orange*) or downregulated genes (*blue*). HO, homozygous knockout; KEGG, Kyoto Encyclopedia of Genes and Genomes; PP2A, protein phosphatase 2A.

To address the role of PP2A in the regulation of liver metabolism, liver tissues from WT and homozygous knockout (HO) mice at the 3 months of age were collected and subjected to transcriptome analysis (n = 3). We identified 2695 differentially expressed genes (DEGs) (mapping to 3453 transcripts), of which 1242 and 1453 were upregulated and downregulated, respectively, in HO mouse with fold change ≥2 or ≤−2 (Fig. 1*A* and Data S1). Gene Ontology analysis showed that most of DEGs were implicated in metabolic-related biological processes, including organic substance metabolic, nitrogen compound metabolic, cellular protein metabolic, and primary metabolic processes (Fig. S1). Using Kyoto Encyclopedia of Genes and Genomes (KEGG) analysis, these DEGs were enriched into 10 biological pathways (*p* < 0.05). Pathways associated with metabolism were the most significantly affected (Fig. 1*B*). Next, the identified DEGs were subjected to Gene Set Enrichment Analysis, and six gene sets were significantly recognized (*p* < 0.05) (Fig. 1*C*). The functional annotation revealed that drug metabolism CYP, drug metabolism other enzymes, steroid hormone biosynthesis, metabolism of xenobiotics by CYP, and retinol metabolism were suppressed, whereas the ubiquitin-mediated proteolysis was activated. Moreover, we noted that the changes in the expression of CYP450 genes were the most pronounced (Fig. 1*C*). Taken together, these findings indicate a critical role of PP2A in regulating the hepatic metabolism.

**Figure 2:**
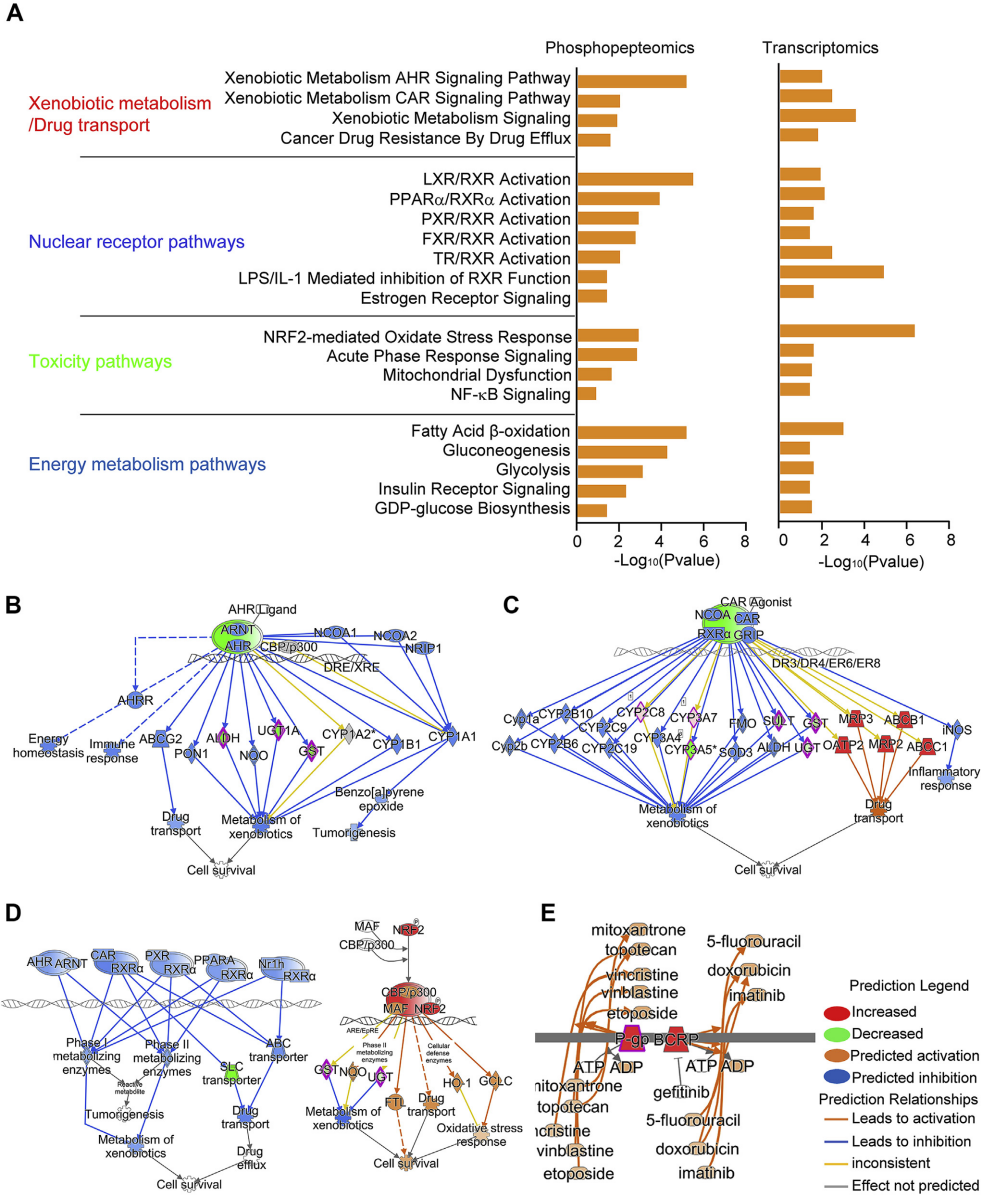
**Identification of pathways underlying liver metabolism regulated by PP2A.***A*, comparative analysis was performed in HO mice by ingenuity pathway analysis (IPA) to explore the pathways based on the differentially expressed genes and hyperphosphorylated proteins. Pathway analysis with *p* < 0.05 was considered significantly enriched. Molecule activity predictor analysis was conducted to predict the effect on the downstream function upon suppression of AHR signaling pathways (*B*), CAR signaling pathways (*C*), xenobiotic metabolism pathways (*D*), or activation of drug transport (*E*). The possible interaction molecules in the network were illustrated based on IPA. AHR, aryl hydrocarbon receptor; CAR, constitutive active androstane receptor; HO, homozygous knockout; PP2A, protein phosphatase 2A.

To clarify the mechanism by which PP2A was involved in the regulation of liver metabolism, we performed phosphoproteome analysis by nano–reversed-phase LC–tandem mass spectrometry (MS/MS), as described previously (28). About 549 upregulated phosphoproteins including 774 phosphorylation sites (log_2_ [fold change] ≥0.26) appeared in liver tissues from HO mice at the age of 3 months (Data S2). Particularly, we focused on the perturbed phosphoproteins that were related to metabolic enzymes, transcription factors, and transporter proteins, as well as those that were consistent with the data from transcriptome analysis (Table S1). Next, we performed comparative analysis to identify the perturbed canonical pathways attributable to PP2A inactivation in two omics datasets. As a result, we identified that 121 and 127 pathways significantly altered with upregulated phosphoproteins and DEGs, respectively (*p* < 0.05) (Data S3). Of the 44 overlapping pathways, 20 pathways were categorized to xenobiotic metabolism/drug transport, nuclear receptor pathways, toxicity pathways, and energy metabolism pathways (Fig. 2*A*). Additional analysis by Molecule Activity Predictor program showed that the pathways underlying xenobiotic metabolisms, including AHR, xenobiotic metabolism, and CAR were significantly suppressed (Fig. 2, *B*–*D*). However, pathways implicated in drug resistance were activated (Fig. 2*E*). Collectively, these results demonstrate that PP2A is involved in the regulation of xenobiotic/drug metabolism and drug transport.

### Characterization of metabolic enzymes regulated by PP2A

**Figure 3:**
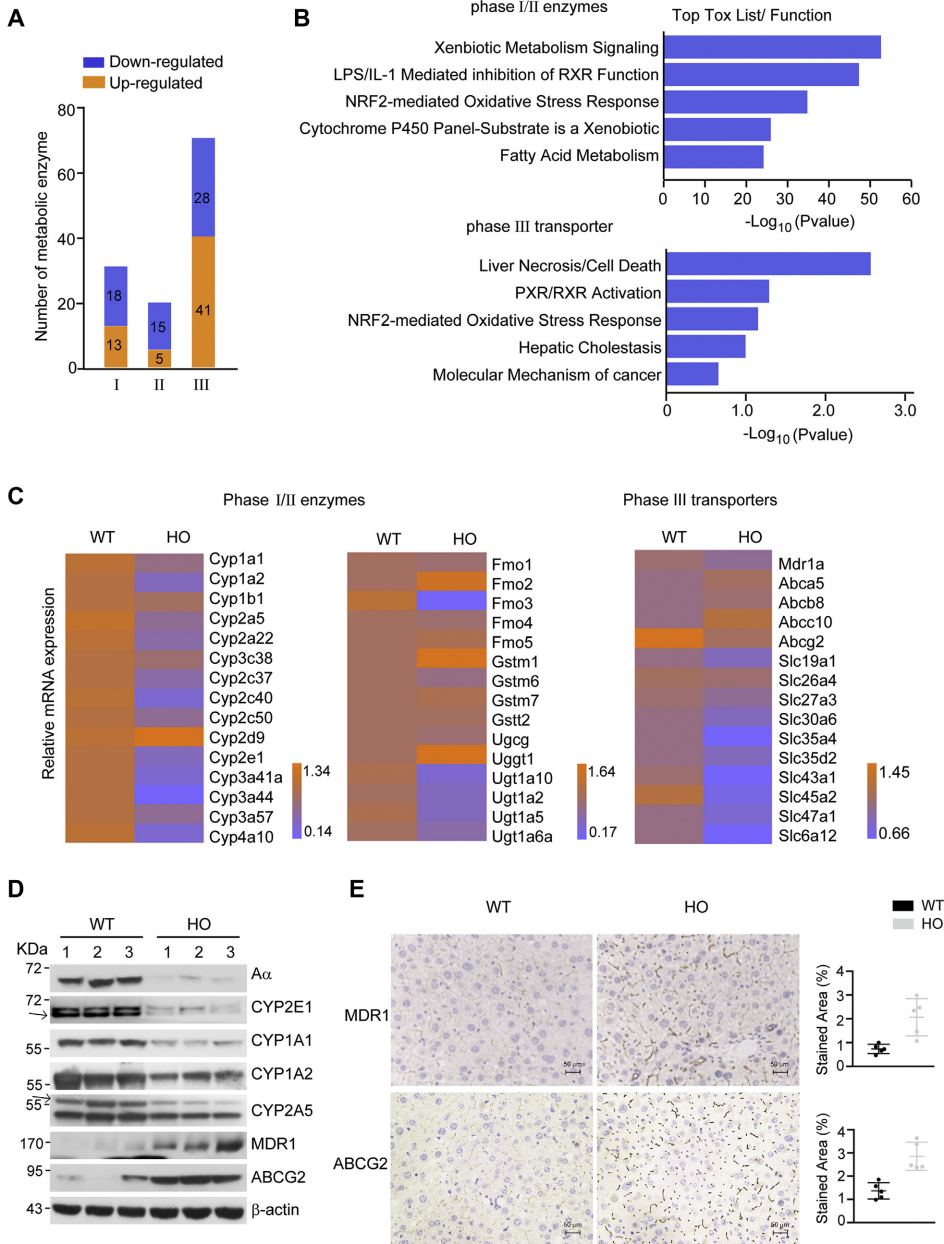
**Identification of metabolic enzymes regulated by PP2A.***A*, the numbers of significantly changed metabolic enzymes including I, II, and III enzymes in transcriptomics and phosphoproteomics are shown as upregulated (*orange*) or downregulated (*blue*) DEGs or phosphoproteins, respectively. *B*, toxicity function analysis was conducted using IPA platform based on the metabolic DEGs in phase I/I and phase III metabolic enzymes, respectively. Toxicity pathway analysis with *p* < 0.05 was considered significantly enriched. *C*, real-time quantitative PCR was performed to verify the mRNA level of 45 metabolic enzyme genes in WT and HO mouse liver tissues (mean ± SD, n = 3). Relative mRNA levels were presented in the heatmap and as fold change relative to WT mice and shown as upregulated (*orange*) or downregulated (*green*) gene expression. *D*, the immunoblotting analysis of indicated proteins in liver tissue from WT and HO mouse. *E*, the representative images were shown for liver sections stained with MDR1 or ABCG2 antibody (400× magnification; the scale bar represents 50 μm). Quantification of MDR1- or ABCG2-stained area expressed as a percentage of liver fields occupied (mean ± SD, n = 5). DEG, differentially expressed gene; HO, homozygous knockout; IPA, Ingenuity Pathway Analysis; PP2A, protein phosphatase 2A.

To profile the metabolic enzymes affected by deletion of PP2A Aα subunit, we analyzed the altered metabolic enzyme genes in HO mouse liver. As shown in Figure 3*A*, we identified 120 metabolic DEGs, with 59 upregulated and 61 downregulated (Data S4). Of these metabolic DEGs, 25.8%, 16.7%, and 57.5% were classified as phase I, II, and III enzymes, respectively. The functional annotation revealed that metabolic DEGs of I/II enzymes were correlated with metabolic disease, liver cirrhosis, liver cholestasis, or liver hyperplasia/hyperproliferation. The top five toxicity pathways enriched were xenobiotic metabolism signaling, LPS/IL-1 mediated inhibition of RXR, NRF2-mediated oxidative stress, cytochrome P450 to xenobiotic, and fatty acid metabolism (Fig. 3*B*). In addition, perturbation in the following five toxicity pathways, liver necrosis/cell death, PXR/RXR activation, NRF2-mediated oxidative stress, hepatic cholestasis, and molecular mechanism of cancer were predicted from DEGs of phase III enzymes (Fig. 3*B*). These results suggest that PP2A is involved in regulation of the cellular responses and liver injuries, at least in part, through the modulation of metabolic enzymes.

Next, we verified 45 metabolic enzymes from the transcriptome data by quantitative PCR. As shown in Figure 3*C*, the mRNA levels of nine or 28 metabolic DEGs were significantly increased (such as Cyp2d9, Fmo2, and Gstm1) or decreased (such as Cyp1a1, Cyp2a5, and Cyp2e1), respectively in HO mouse livers compared with those in WT littermates, 73.3% of which were consistent with transcriptome analysis. In general, depletion of PP2A Aα subunit in hepatocytes led to decreased expression of solute carrier subfamilies and Cyp450 subfamilies but increased expression of ABC subfamilies. Notably, we validated 60 metabolic DEGs at the protein expression level by proteomics analysis (Data S5). In addition, protein levels of CYP2E1, CYP1A1, CYP1A2, CYP2A5, MDR1 (encoded by multi-drug resistance gene 1), and ABCG2 were further confirmed by immunoblotting or immunohistochemistry analysis (Fig. 3*D*). The results showed that MDR1- and ABCG2-positive stainings were increased by 1.48- and 1.04-fold, respectively, in HO mice compared with that in WT mice (Fig. 3*E*).

We assessed whether PP2A activation affected the expression of metabolic enzymes. Primary hepatocytes from WT mouse were isolated and cultured for 4, 24, and 48 h, respectively (Fig. S2*A*). The protein levels of CYP2E1, CYP1A1, CYP1A2, and CYP2A5 in cultured primary hepatocytes were decreased in a time-dependent manner (Fig. S2*B*). First, we treated primary mouse hepatocytes with 0, 1, and 2 μM FTY720, an agonist of PP2A, respectively. As a result, FTY720 treatment led to a significant decrease in Cyp1a1, Cyp1a2, Cyp2e1, and Cyp2a5 enzyme mRNA level in primary hepatocytes isolated from WT mice (Fig. S2*C*). This was further confirmed by enzyme activity and immunoblotting analysis (Fig. S2, *D* and *E*). However, FTY720 treatment failed to affect the enzyme expression in hepatocytes isolated from HO mice (Fig. S2*D*), suggesting that PP2A enzyme activity is critical for maintaining liver metabolic enzymes. Taken together, these results indicate that the regulatory role of PP2A in xenobiotic/drug metabolism was distinct toward different phases of metabolic enzymes.

### PP2A was involved in regulation of chemical-induced hepatotoxicity through modulating the metabolic enzyme expressions

Xenobiotic-induced toxicity depends predominantly on biotransformation, which is mediated by metabolic enzymes. Next, we investigated the effect of PP2A inactivation on the induction of metabolic enzymes and hepatotoxicity induced by various chemicals or drugs. Primary hepatocytes isolated from WT or HO mice were subjected to treatment with 15 different chemicals or drugs, respectively. As a result, the treatment of benzo(a)pyrene (BaP), 3-methylcholane, pyrazole, ethanol, acetaminophen, adriamycin (ADR), methotrexate, or 5-fluorouracil led to a decreased cell viability in a dose-dependent manner but greatly attenuated in hepatocytes from HO mice (Fig. S3*A* and Table S2). Induction of Cyp1a1, Cyp1b1, Cyp1a2, Cyp2b10, Cyp3a11, Cyp2a5, Cyp2e1, and Cyp3a41 expression was seen in hepatocytes from WT mice treated with 20 μM BaP, 5 μM 3-methylcholane, 1 mM phenobarbital, 10 μM rifampicin, 10 μM pyrazole, 50 mM ethanol, or 5 μM amiodarone, respectively. However, PP2A Aα deletion led to a decrease in the induction of these metabolic enzymes (Fig. S3*B*). In addition, 5-fluorouracil, cisplatin, or paclitaxel treatment had no effect on mRNA expression of Mdr1a, Abcc3, or Abcg2 (Fig. S3*C*) but led to an increase at the protein level (Fig. S3*D*). Notably, the induction of transporter protein (phase III enzymes) in HO mouse livers was more profound. Taken together, these observations suggest that PP2A might play a critical role in determining chemical-induced hepatotoxicity through both transcriptional and PTMs of metabolic enzymes.

**Figure 4:**
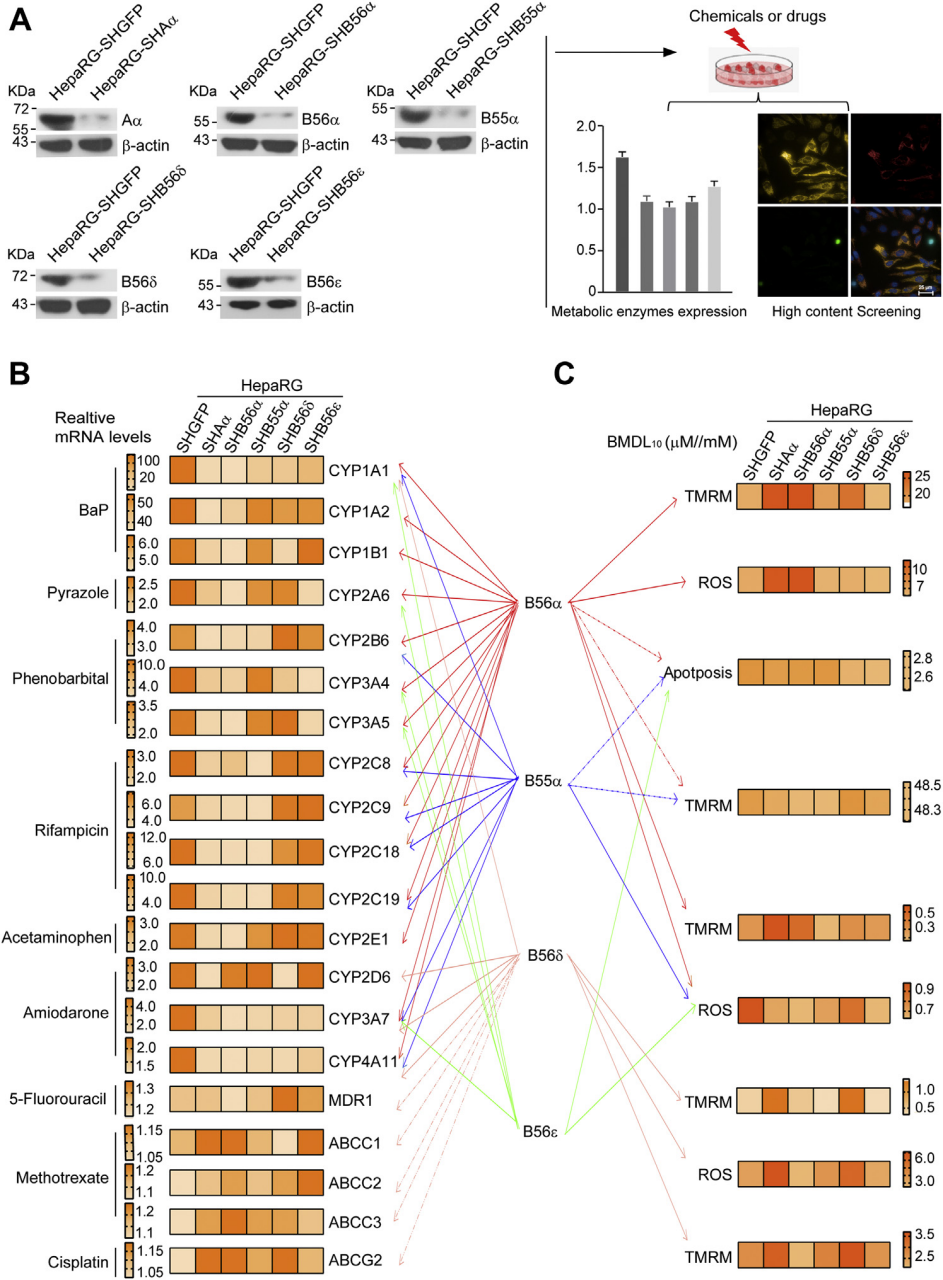
**Specific PP2A holoenzyme was involved in regulating chemical-induced hepatotoxicity.***A*, HepaRG cells were transfected with vectors encoding shRNAs targeting GFP, Aα, B55α, B56α, B56δ, and B56ε, respectively, to generate stable cell lines as indicated. The protein levels were determined by immunoblotting analysis with the indicated antibodies. The cell lines were treated with 15 chemicals or drugs and followed by high-content screening assays and the measurement of metabolic enzyme expression. Schematic diagram showed the fold change of metabolic enzyme induction (*B*) and the BMDL_10_ of indicated cytotoxicity end point (*C*) and for indicated chemicals or drugs in HepaRG cells expressing shAα, shB55α, shB56α, shB56δ, or shB56ε. The interactions among indicated metabolic enzymes, cytotoxicity, and PP2A B subunit were labeled with *arrows*. *Dotted arrows* represent no obvious cytotoxicity or mRNA changes in response to indicated chemical treatment. BMD, benchmark dose; PP2A, protein phosphatase 2A.

Next, we addressed which specific PP2A complex was responsible for the modifications of metabolic enzymes. Given the abundance and stability of CYP in HepaRG cells (29), we first verified that differentiated HepaRG cells displayed high albumin production and a significant increase in Cyp3a4, Cyp1a1, and Cyp1a2 mRNA compared with that in undifferentiated HepaRG cells (*p* < 0.05) (Fig. S4*A*). Since B55α, B56α, B56ε, and B56δ subunits were dramatically decreased in HO mouse liver (28), we speculated that the altered metabolic enzymes might be mediated by the dysregulation of specific B subunit–mediated dephosphorylation. We generated HepaRG cell lines stably expressing shRNAs, shB55α, shB56α, and shB56ε, or shB56δ (Fig. 4*A*) and examined the induction of 20 metabolic enzymes in cells upon the suppression of specific B subunit in response to treatment of various chemicals or drugs (Table S3). Notably, the suppression of Aα or B56α subunit led to a significant decline in the induction of metabolic enzymes, such as Cyp1a1, Cyp1a2, Cyp1b1, Cyp2e1, Cyp2a6, Cyp2b6, Cyp3a4, Cyp3a5, Cyp2c8, Cyp2c9, Cyp2c18, Cyp2c19, Cyp3a7, or Cyp4a11 (Fig. 4*B*). Furthermore, the suppression of Aα or B55α subunit resulted in a decline in the induction of metabolic enzymes, including Cyp1a1, Cyp2b6, Cyp2c8, Cyp2c9, Cyp2c18, Cyp2c19, Cyp3a7, and Cyp4a11. In addition, the mRNA level of five transport proteins remained unchanged when HepaRG cells were treated with various chemicals or drugs. The family subtypes of CYP in human and mouse are shown in Table S4. Concomitantly, the biological end points including cell viability, apoptosis, mitochondrial membrane potential (MMP), and reactive oxygen species (ROS) generation were determined by high-content imaging assay. The value of benchmark dose (BMD)L_10_ was used as a threshold for determining toxicity in response to xenobiotic treatments (30). We found that the sensitivity of these biological end points varied among different chemicals. Particularly, mitochondrial damage was the most sensitive marker in response to treatment with BaP, acetaminophen, 5-fluorouracil, and cisplatin (Table S5). Moreover, HepaRG-SHAα, HepaRG-SHB56α, and HepaRG-SHB56δ cells displayed attenuated mitochondrial damage or apoptosis upon treatment with most of the chemicals or drugs (Fig. 4*C* and Table S6). Notably, these PP2A B subunits varied in the extent of cytotoxicity in response to the various chemicals or drugs. For instance, the attenuated mitochondrial damage induced by BaP was the most profound in HepaRG-SHB56α cells (Figs. 4*C* and S4*B*). In parallel, B56δ suppression led to a dramatic decrease in mitochondrial damage induced by 5-fluorouracil (Figs. 4*C* and S4*C*). Collectively, these results suggest that dysregulation of metabolic enzymes because of the lack of specific PP2A holoenzymes confers cells with sensitivity to xenobiotic-induced hepatotoxicity.

### The regulatory mode of metabolic enzymes by specific PP2A complexes

**Figure 5:**
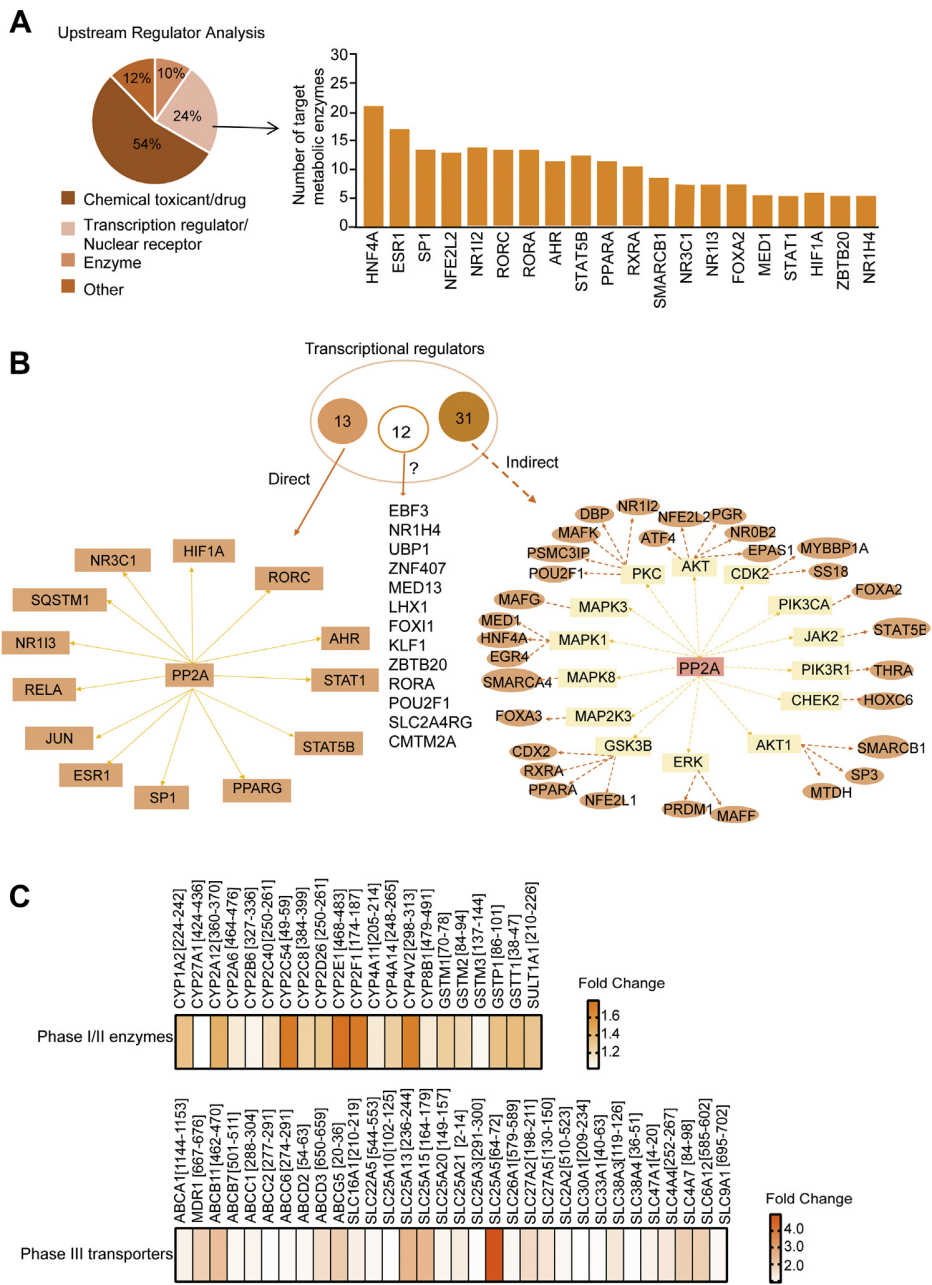
**The mode of action by which PP2A regulated the expression of metabolic enzymes.***A*, the upstream regulators for the metabolic DEGs were predicted by IPA program. The percentage of upstream regulators was shown in a pie chart. The numbers of metabolic enzymes targeted by top 20 transcriptional regulators were shown. *B*, the predicted associations between PP2A and 56 transcription factors with specific Ser/Thr phosphorylation sites. The schematic diagram illustrated candidate phosphorylated transcription factors regulated by PP2A directly or indirectly. *C*, heatmap of hyperphosphorylated metabolic enzymes and respective phosphorylation sites in HO mice relative to WT mice. DEG, differentially expressed gene; HO, homozygous knockout; IPA, Ingenuity Pathway Analysis; PP2A, protein phosphatase 2A.

To elucidate the mechanism by which PP2A regulated the activity of metabolic enzymes, we conducted bioinformatic analysis to predict the upstream modulators for metabolic DEGs using Ingenuity Pathway Analysis (IPA) software (Ingenuity Systems, Inc). As a result, 456 upstream factors were identified to be involved in regulating these metabolic DEGs. Of these upstream factors, 24% was classified as transcriptional regulators and nuclear receptors (Fig. 5*A* and Data S6). Particularly, HNF-4α, ESR1, SP1, NFE2L2, NR1I2, RORC, RORα, AHR, STAT5B, or peroxisome proliferator–activated receptor alpha ranked at the highest in terms of numbers of target metabolic enzyme genes. Notably, 46 of 56 transcriptional regulators could be phosphorylated at serine/threonine residue predicted by Phospho.ELM program (http://phospho.elm.eu.org) (Fig. 5*B*). Importantly, these 44 transcriptional regulators have been previously reported to be regulated by PP2A (Fig. 5*B*). IPA predicted that the remaining 12 transcriptional regulators might potentially be the regulatory targets of PP2A (Fig. 5*B*). These results suggest that PP2A might regulate the expression of metabolic enzymes *via* dephosphorylation of transcription factors. Subsequent phosphoproteome analysis showed the enhanced phosphorylation of 53 metabolic enzymes upon the deletion of PP2A Aα subunit (Fig. 5*C*). In addition, 30.2% of these enzymes displayed changes in phosphorylation status coupling with altered protein levels, suggesting that the dephosphorylation of these transcription factors might be a critical mode of action for the modulation of metabolic enzymes. Furthermore, IPA predicted that PP2A B55α, B56α, B56δ, or B56ε holoenzyme might be involved in the regulation of various metabolic enzymes (Data S7). Of note, PP2A B55α subunit participated in the regulation of drug metabolic enzymes, such as Cyp3a4, Cyp2c8, or Cyp2c9. PP2A B56α was mainly involved in the xenobiotic metabolism–related metabolic enzymes, such as Cyp1a1, Cyp1a2, or Cyp2e1. In addition, PP2A B56δ was predicted to regulate several phase III enzymes, including ABCD, ABCB, ABCC, and ABCG subfamilies. Therefore, these findings suggest that specific PP2A subunit determines the regulatory role of PP2A in liver metabolism. Collectively, specific PP2A complexes regulate the metabolic enzyme induction through dephosphorylation of transcription factors or target enzymes, which enables hepatocytes for rapid adaption to environmental stress.

### PP2A B56α–AHR–Cyp1a1 axis is involved in regulation of BaP-induced cytotoxicity

**Figure 6:**
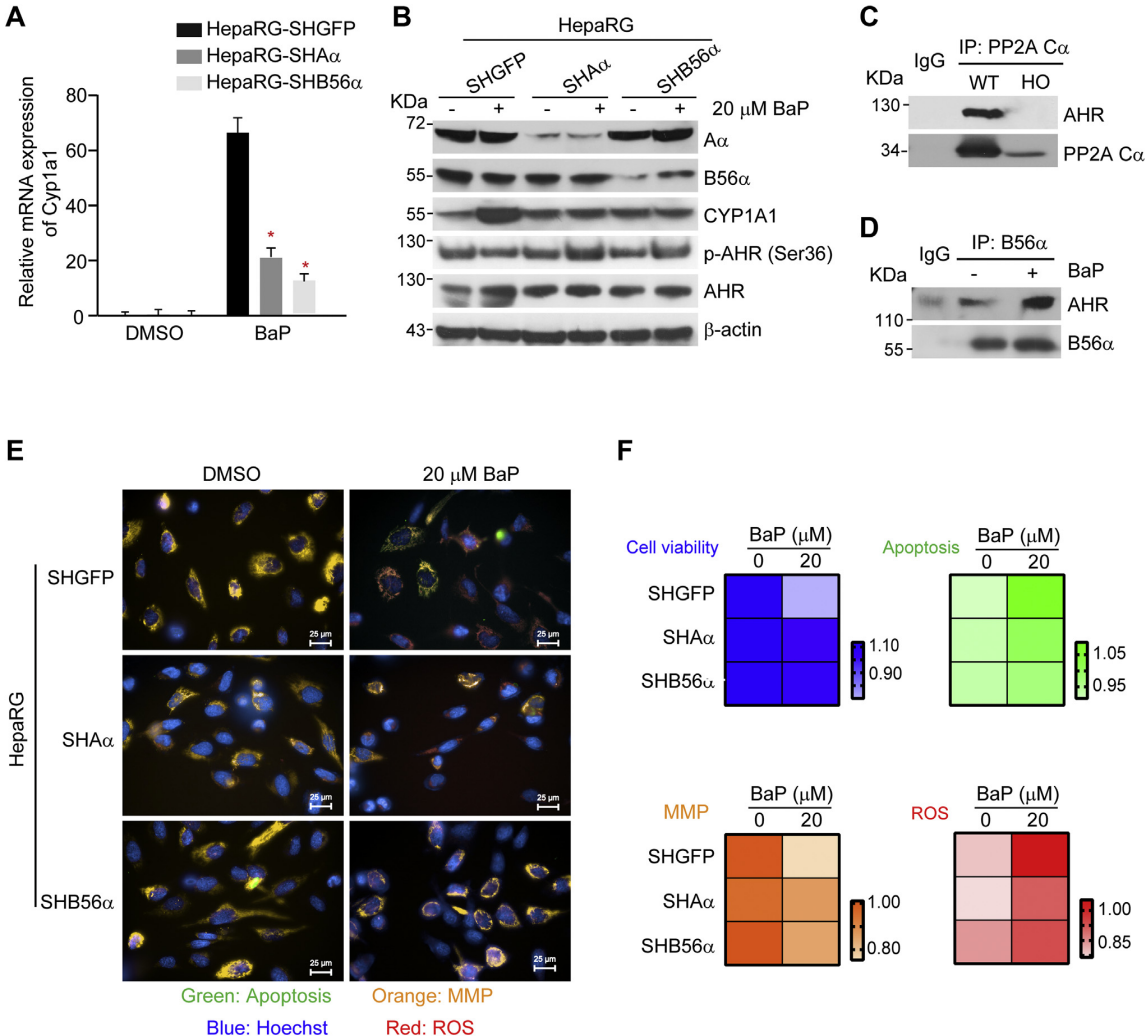
**Specific PP2A B56α complexes participated in BaP-induced metabolic activation and cytotoxicity.** HepaRG-SHGFP, HepaRG-SHAα, and HepaRG-SHB56α cells were treated with 20 μM BaP for 24 h. *A*,the relative mRNA levels of Cyp1a1. *B*, immunoblotting analysis was performed to detect the protein levels in indicated cell lines. *C*, co-IP assays were performed with an antibody against PP2A Cα and followed by immunoblotting with antibodies against indicated proteins. *D*, co-IP analysis of HepaRG cells treated with 20 μM BaP was performed with antibody against B56α subunit and followed by immunoblotting with AHR and B56α antibodies. *E*, the high-content screening analysis was performed in indicated cells stained with apoptosis (*green*), mitochondrial damage (*orange*), and ROS (*red*). The nuclei (*blue*) were stained with DAPI. *F*, the quantification was conducted based on fluorescence intensity for the indicated end point. AHR, aryl hydrocarbon receptor; BaP, benzo(a)pyrene; co-IP, coimmunoprecipitation; DAPI, 4,6-diamidino-2-phenylindole; PP2A, protein phosphatase 2A; ROS, reactive oxygen species.

As described previously, the change of Cyp1a1 expression affected by PP2A activation was the most profound. To validate the reliability of regulatory mode, we performed functional assays and analysis of protein interactions in representative regulatory axes. First, we assumed a possible regulatory axis of specific PP2A complex–phosphorylated AHR (p-AHR)–Cyp1a1. To test this hypothesis, we treated HepaRG cells expressing shRNAs targeting distinct PP2A subunit with 20 μM BaP, an agonist of AHR signal pathway. In agreement with the results from primary mouse hepatocytes, Cyp1a1 induction declined by 68.5% and 71.7%, respectively, in HepaRG-SHAα or HepaRG-SHB56α cells following BaP treatment (Fig. 6*A*). Correspondingly, the p-AHR at serine 36 was decreased by 64.2% in HepaRG-SHGFP cells treated with BaP. In contrast, we observed an enhanced p-AHR at serine 6 and a decreased induction of Cyp1a1 in HepaRG-SHB56α and HepaRG-SHAα cells upon BaP treatment (Fig. 6*B*), indicating that PP2A might be responsible for dephosphorylation of p-AHR. Coimmunoprecipitation (co-IP) assay confirmed the interaction between Cα subunit and AHR (Fig. 6*C*) and between B56α subunit and AHR in HepaRG cells (Fig. 6*D*). In agreement with these observations, cell viability was decreased by 29.8% in HepaRG-SHGFP cells treated with 20 μM BaP. However, this effect was attenuated by 72.5% and 77.2%, respectively, in HepaRG-SHB56α and HepaRG-SHAα cells (Fig. 6, *E* and *F*), indicating the involvement of PP2A B56α complexes in regulating BaP-induced cytotoxicity. Similar results were found with other toxicity end points, including apoptosis, MMP, and ROS generation. In particular, the effects of PP2A B56α suppression in attenuating mitochondrial damage were the most profound. These results confirmed that the regulatory axis of PP2A B56α–AHR–Cyp1a1 was implicated in mediating BaP-induced cytotoxicity. Taken together, PP2A plays an important role in regulation of the transcriptional activation of metabolic enzymes through dephosphorylation of the key transcription factors, demonstrating a key mode of action in metabolism in response to environmental stresses.

### PP2A B56δ complex dephosphorylates MDR1 and confers cells with drug resistance

**Figure 7:**
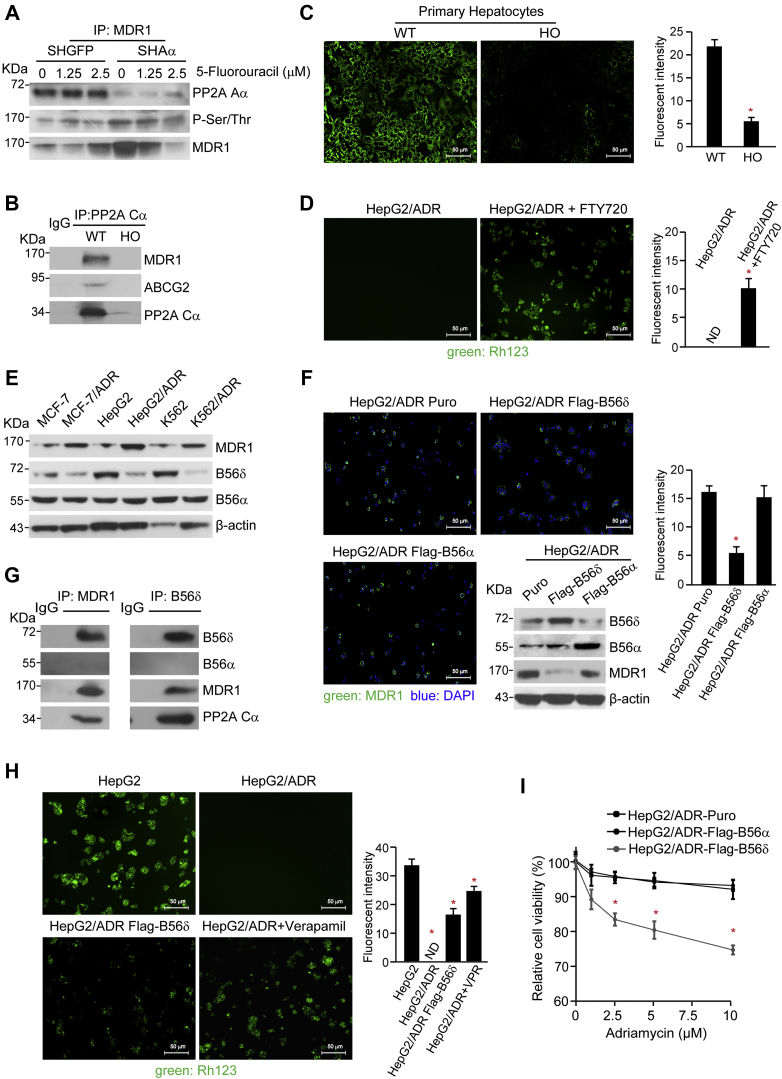
**Specific PP2A complexes containing B56δ directly regulated the activity of MDR1.***A*, HepaRG-SHGFP and HepaRG-SHAα cells were treated with 5-fluorouracil for 24 h. About 3 mg of the cell lysates were subjected to co-IP with an antibody against MDR1 and followed by immunoblotting analysis with specific antibodies indicated. *B*, co-IP analyses were performed in liver tissue from WT and HO mice with an antibody against B56α subunit and followed by immunoblotting with antibodies against MDR1, ABCG2, and PP2A Cα. Immunofluorescence analysis was performed in primary hepatocytes from WT and HO mouse (*C*) and HepG2/ADR cells with or without FTY720 treatment (*D*). Quantification of mean fluorescence intensity expressed as a ratio of integrated density to area of fields (mean ± SD). ∗*p* < 0.05, compared with the corresponding control cells. *E*, protein levels of MDR1, B56α, and B56δ in indicated cell lines were determined by immunoblotting analysis. *F*, HepG2/ADR cells were transfected with vectors encoding FLAG-tagged B56α or B56δ to generate stable cell lines as indicated. Protein levels were determined by immunoblotting analysis with the indicated antibodies. The immunofluorescence analysis of these cells stained with an antibody against MDR1 (*green*). The nuclei (*blue*) were stained with DAPI. *G*, co-IP analyses were conducted in HepG2/ADR cells with antibodies against MDR1 and B56δ and followed by immunoblotting with indicated antibodies. *H*, the accumulation of Rh123 in HepG2, HepG2/ADR, HepG2/ADR-FLAG-B56δ, and HepG2/ADR cells treated with verapamil was determined by immunofluorescence analysis. *I*, HepG2/ADR cells expressing control vector, FLAG-B56α, or FLAG-B56δ were treated with ADR at indicated concentrations. MTT assay was applied to examine the relative cell viability. ∗*p* < 0.05 compared with HepG2/ADR-control cells. ADR, adriamycin; co-IP, coimmunoprecipitation; DAPI, 4,6-diamidino-2-phenylindole; HO, homozygous knockout; MTT, 3-(4,5-dimethylthiazol-2-yl)-2,5-diphenyl-2H-tetrazolium bromide; PP2A, protein phosphatase 2A; Rh123, rhodamine 123.

Since PP2A was predicted to regulate the expressions of metabolic enzymes by dephosphorylation of transporter proteins, next, we examined the phosphorylation status of MDR1 in cells expressing shAα (HepaRG-SHAα cells). As a result, 5-fluorouracil treatment led to an enhanced phosphorylation level of MDR1 in HepaRG-SHAα cells (Fig. 7*A*), whereas the mRNA levels remained unchanged. In contrast, we hardly detected the phosphorylation of MDR1 in hepatocytes isolated from WT mice. Importantly, the co-IP assay revealed that MDR1 was in complex with PP2A Cα subunit (Fig. 7*B*), indicating that PP2A was directly involved in regulation of MDR1. In agreement to these observations, the functional assay revealed that the content of rhodamine 123 (Rh123) accumulated in hepatocytes was four-fold higher in WT mice than HO mice (Fig. 7*C*), indicating that phosphorylation of MDR1 led to a faster efflux attributable to enhanced transporter activity of MDR1. In particular, activation of PP2A resulted in increased Rh123 accumulation in human hepatocarcinoma cells resistance to ADR (HepG2/ADR cells) (Fig. 7*D*). Collectively, these results demonstrate that PP2A is involved in regulation of MDR1 activity by direct dephosphorylation, conferring cells with drug resistance.

Next, we determined which PP2A complex participated in the regulation of MDR1. Immunoblotting analysis showed the significant increase of MDR1 protein levels in three types of ADR-resistant cell lines, including MCF-7/ADR, HepG2/ADR, and K562/ADR, compared with corresponding ADR-sensitive cells. As suppression of B56δ or B56α attenuated the cytotoxicity induced by 5-fluorouracil, we determined the expression of B56δ and B56α subunit in these ADR-resistant tumor cell lines. B56δ expression declined while no obvious change was noted in B56α expression (Fig. 7*E*). Next, we generated stable HepG2/ADR cells overexpressing B56δ or B56α subunit (with FLAG tagged). Immunofluorescence analysis showed that B56δ overexpression led to a downregulation of phosphorylated MDR1 and the increased protein levels (Fig. 7*F*). In contrast, we failed to observe these effects in cells overexpressing B56α subunit. Although MDR1 expression was hardly detected in HepG2 cells, the suppression of B56δ remarkably upregulated the MDR1 protein as determined by both immunofluorescence and immunoblotting analyses (Fig. S5, *A* and *B*), implicating a role of B56δ subunit in modulating MDR1 expression. Importantly, co-IP assay confirmed the interaction between B56δ subunit and MDR1 (Fig. 7*G*), indicating that PP2A-B56δ holoenzyme might be responsible for MDR1 dephosphorylation.

Previous studies have reported that phosphorylation was essential for ubiquitination-mediated degradation of MDR1. Thus, we speculated that PP2A-B56δ holoenzyme might be involved in regulation of MDR1 protein stability. To test this hypothesis, we overexpressed B56δ subunit or treated HepG2/ADR cells with verapamil, an MDR1 inhibitor, and found an increasing accumulation of Rh123 in HepG2/ADR cells (Fig. 7*H*), indicating that B56δ-containing holoenzyme takes part in the reversal of drug resistance. Correspondingly, the cytotoxicity was increased by 17.7% in HepG2/ADR-FLAG-B56δ cells compared with that in HepG2/ADR-puro cells in response to 10 μM ADR treatment (Fig. 7*I*). In contrast, we failed to observe similar effects in HepG2/ADR-FLAG-B56α cells upon ADR treatment. Collectively, these results reveal that PP2A B56δ holoenzyme was indispensable for regulating MDR1 stability and activity, demonstrating a potential role of PP2A B56δ holoenzyme in drug resistance.

## Discussion

**Figure 8:**
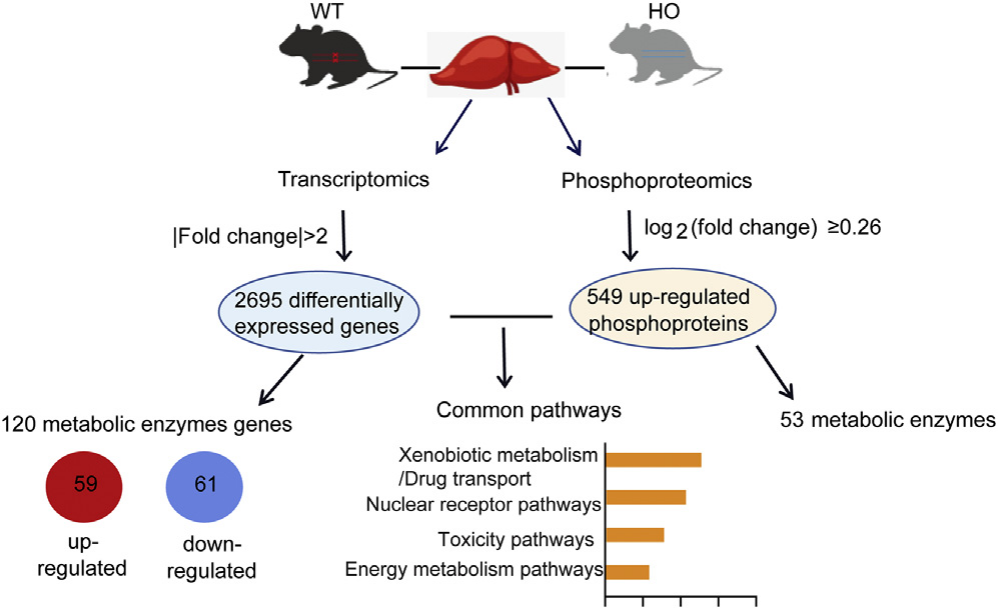
**The schematic overview of transcriptome and phosphoproteome analysis results**.

The understanding of the regulatory mechanism by which xenobiotic-induced modifications of metabolic enzymes was critical for the prediction of consequent pharmacological and toxicological effects. In this study, we integrated the transcriptome and phosphoproteome analysis results (summarized in Fig. 8) and characterized the regulatory role of PP2A in xenobiotic metabolism and hepatotoxicity. We showed two modes of action that PP2A regulated the metabolic enzyme expression through dephosphorylation of transcription factors or nuclear receptors. Particularly, PP2A dephosphorylated and modulated the protein level of metabolic enzymes. Specific PP2A B56α complexes participated in AHR-mediated induction of Cyp1a1, leading to decreased cytotoxicity induced by BaP. PP2A B56δ complexes regulated the stability of MDR1 by direct dephosphorylation, conferring drug resistance. These findings uncover the novel role of PP2A in hepatic metabolism, which is implicated in regulation of environmental pollutant–induced adverse health effects.

In this novel study, we identified the metabolic enzymes regulated by PP2A in a mouse model with hepatocyte-specific deletion of *Ppp2r1a* gene. Moreover, we revealed that PP2A activation could preserve the expression of metabolic enzymes in cultured mouse primary hepatocytes, demonstrating the involvement of PP2A in maintaining metabolic enzymes. Nevertheless, the mode of action with respect to the regulation of metabolic enzymes by specific PP2A complexes has yet to be defined. It is well established that the expressions of metabolic enzymes were mainly regulated by nuclear receptors or transcription factors (31). Moreover, phosphorylation appeared to occur at a motif located in nuclear receptors to transduce xenobiotic cellular signaling that regulated energy metabolism, drug metabolism, or cell proliferation (32). Here, we showed a regulatory pattern of PP2A-mediating alterations in expression of metabolic enzymes in response to exposure of xenobiotics or drugs through dephosphorylation of transcription factors/nuclear receptors. In agreement, previous studies demonstrated that dephosphorylation of CAR by PP2A can be served as a crucial signal for its release from the cytosolic complex upon activator treatment (33, 34). The PP1–PP2A inhibitor, okadaic acid, abolished the activity of pregnant X receptor, leading to the suppression of Cyp3a4 induction (18). In addition, dephosphorylation of Sp1 at serine 59 by PP2A was required for induction of Cyp1a1 transcription in response to 2,3,7,8-tetrachlorodibenzo-p-dioxin or omeprazole (35). Collectively, these observations support that PP2A regulates the transcriptional activation of metabolic enzymes *via* the dephosphorylation of transcription factors.

In addition, we here revealed another mode of action by which PP2A regulated metabolic enzymes *via* dephosphorylation of target enzyme. Prior studies have demonstrated that PTMs, including phosphorylation, ubiquitination, and glycosylation, might play an important role in dynamic regulation of drug transporters (36). Although post-translational regulation is not predominant in regulation of phase I enzymes, it has been reported that CYP2E1 was regulated mainly at the post-translational level in response to xenobiotics, such as ethanol, acetone, pyrazole, and isoniazid (37). Here, we identified in HO mice several differentially regulated phosphorylated metabolic enzymes, particularly transporters (phase III enzymes). Importantly, we showed that PP2A modulated the metabolic enzymes by dephosphorylation, thereby altering environmental pollutant–induced or drug-induced biological consequences. Although two regulatory modes of action have been proposed in this study, at the transcriptional and post-translational levels, some of these enzymes might be regulated at both regulatory modes of action. For instance, Cyp2e1 expression could be transcriptionally regulated by multiple transcription factors including JUN, HNF-4α, and β-catenin (27, 38, 39). Alternatively, isoniazid induction of Cyp2e1 could be involved in alteration of protein stability (40). In agreement with these findings, we showed that an enhanced phosphorylated Cyp2e1 in the phosphoproteome dataset at serine 129 and PP2A-mediated dephosphorylation of β-catenin was involved in suppression of Cyp2e1 expression (27). Together, these findings provide evidence that PP2A regulated the expression of Cyp2e1 through modulating its phosphorylation status. Similar regulatory mode appears in modification of Mdr1 gene. The expression of Mdr1 could be transcriptionally activated by Sp1 in response to UV irradiation (41). In addition, CAR was found to be a regulator of MDR1 expression, allowing CD4^+^ T cells safeguard against bile acid toxicity and inflammation in mouse small intestine (42). Although we found that the regulation of MDR1 seemed to be at the protein level, further characterizations are warranted to understand how these regulatory modes of action coordinated in modification of metabolic enzymes in response to environmental stimuli.

In this study, we noted that a group of phase III enzymes were dramatically altered in HO mouse liver at phosphorylation status or protein level. Phase III enzymes were predominantly ABC and solute carrier transporters, which facilitated the entry and elimination of endogenous and xenobiotic compounds (12). MDR1/ABCB1, a member of the ABC group of proteins, was well known for the significance in clinical therapeutics. MDR1 has been characterized as a central therapeutic target to combat drug resistance (43). A prior study has shown that Pim-1 kinase phosphorylated MDR1 and thereby protected core-glycosylated MDR1 from proteasomal degradation, also enabled MDR1 glycosylation and cell surface trafficking of glycosylated MDR1 (20). These findings indicated that phosphorylation was an important mechanism underlying the protein stability of MDR1. Here, we demonstrated that MDR1 was directly dephosphorylated by PP2A B56δ. These observations suggest that a particular PP2A subunit may be a promising target for drug resistance. Activation of PP2A B56δ might be a novel approach to abrogate MDR1-mediated drug resistance by promoting MDR1 degradation.

In summary, we reveal the important role of PP2A in regulating metabolic enzyme expression, and specific PP2A complexes are involved in regulation of metabolic enzymes at both transcriptional and post-translational levels through dephosphorylation of the targets. Our findings shed light on the understanding of protein phosphatase regulating liver metabolism and cellular functions in response to environmental stress.

## Experimental procedures

### Primary mouse hepatocyte and HepaRG cell culture

Mouse model with hepatocyte-specific deletion of *Ppp2r1a* (encoding PP2A Aα subunit) gene (HO) and matched WT mouse was generated as described previously (28). In detail, mice with hepatocyte-specific deletion of *Ppp2r1a* were generated by crossing *Ppp2r1a*^*loxp/+*^ mice with *Alb*-Cre mice (genetic background was C57BL/6). *Ppp2r1a*^*loxp/+*^ mice with genetic background of FVB.129S were purchased from the Jackson Laboratory (strain 017441), which contained loxP sites flanking exons 5 to 6 of the *Ppp2r1a* gene. Primary hepatocytes were isolated from WT or HO female mice at the age of 3 months by two-step collagenase perfusion (44). The primary cultures were maintained in Williams’ medium E supplemented with 10% fetal bovine serum, 4 μg/ml insulin, 1 μM dexamethasone, 1% penicillin/streptomycin, 15 mM Hepes, 2 mM l-glutamine. Upon cell attachment, the medium containing the unattached cells and debris was removed, and the cultures were coated with a second collagen layer to form a collagen–collagen sandwich configuration. HepaRG cells (human hepatocarcinoma–derived cell line) were purchased from Thermo Fisher Scientific. For induction of differentiation, HepaRG cells were incubated in Williams' E medium containing 1.7% dimethyl sulfoxide for 14 days. All animal protocols were approved by the Animal Care and Use Committee of the Model Animal Research Center of Sun Yat-sen University.

### Establishments of stable cell lines

The shRNAs against specific PP2A subunit were generously provided by Dr William C. Hahn (Harvard Medical School). Considering the abundance of metabolic enzymes in AML12 cells (mouse immortalized liver cell line) and the feasibility of gene suppression on primary mouse hepatocyte, we chose HepaRG cells or HepG2/ADR cells to explore the role of specific PP2A subunits. To generate stable cell lines, the pLKO.1-puro shGFP and pLKO.1-puro vectors containing shRNA targeting Aα, B56α, B55α, B56δ, or B56ε were introduced into HepaRG cells, HepG2 cells, or HepG2/ADR cells, respectively, by lentivirus infection and selected with puromycin (1 μg/ml).

### Phosphoproteome and transcriptome analysis

Liver tissues from female WT and HO mice (n = 3) at the 3 months of age were collected and subjected to nano reversed-phase LC–electrospray ionization–MS/MS analysis as previously described (45). Raw data were searched in Mascot (2.3.2) (Matrix Science) for automated peptide identification using mouse UniProtKB (www.uniprot.org, 201,705) proteome sequence databases, and the parameters were set as described previously (46). Peptide modification of phosphor S, T, Y (+79.966 Da) was included for assigning phosphorylated residues. The MS/MS were acquired in the orbitrap mass analyzer with a mass resolution of 17,500 at *m/z* 200 for identification of the adsorbed proteins. RNA sequencing was performed on an Illumina HiSeq platform. Data were acquired with Illumina BeadChip Reader and evaluated using Illumina BeadStudio Application by Genergy Bio. The raw reads were sorted out by deleting reads with low-quality sequence (quality value <30). The expression level of gene was normalized by fragments per kilobase per million). DEGs were defined with the following screening criteria: absolute log_2_ (fold change) ≥1 and *p* < 0.05 in mouse liver tissues. The upregulated phosphoproteins (differentially expressed phosphoproteins [DEPPs]) were selected with the thresholds log_2_ (fold change) ≥0.26.

### Bioinformatics analysis and functional annotation

The analysis of DEPPs or genes was conducted with IPA software. Ingenuity knowledge–based tool was used to identify significant canonical pathways and characterize the diseases and functions for the DEGs and DEPPs. Pathways were considered significantly enriched when *p* < 0.05. In addition, DEGs were subjected to analyses for identifications of upstream regulators, adverse effect prediction, and interaction network.

Gene Ontology, KEGG enrichment analyses, and Gene Set Enrichment Analysis were performed using the clusterProfiler package in R, version 4.1.0. “Mm.h.all.v7.1” and “Mm.c2.cp.kegg.v7.1” entrez.gmt were chosen as the gene set database. A gene set is a group of genes that share pathways, functions, chromosomal localization, or other features. The pathways were considered significantly enriched when *p* < 0.05.

### Examination of cytotoxicity

Primary hepatocytes were seeded into 96-well plates with a density of 1 × 10^4^ per well. About 24 h after seeding, the cells were treated with various chemicals for 24 h. As the subtype variability of phase II enzymes was remarkable, we mainly selected the agonists including 15 chemicals or drugs that were corresponding to the induction of phase I enzymes and the transporters (phase III enzymes). The reference dose for metabolic enzyme induction of each chemical or drug was derived from ToxCast/Tox21 of Environmental Protection Agency. The cell viability was determined by using Cell Proliferation Assay Kit (Promega).

### High-content analysis

HepaRG cells were seeded on 96-well black plates with transparent bottom (PerkinElmer) at a density of 8 × 10^3^ per well. About 24 h after deeding, the HepaRG cells were treated with drugs or chemicals for 24 h, followed by incubation with 100 μl mixed fluorescent dye (Invitrogen) including ethidium homodimer-1 (1 μM), Image-iT TMRM (1×), and CellROX Deep Red Reagent (5 μM) for 30 min. The cultures were washed with PBS for three times and stained with Hoechst 33342 (5 μg/ml). Next, the living-cell imaging was examined under the Operetta CLS Cell Imaging System (PerkinElmer) at 63× objective. Images were analyzed by the cytotoxicity module (PerkinElmer). With these three fluorescent dyes, we were able to quantitatively examine different end points including apoptosis, MMP, and ROS, respectively.

### BMD modeling

The BMD modeling was employed to analyze the dose–response effects on cytotoxicity induced by xenobiotics or drugs (USEPA 2018; Benchmark Dose Software [BMDS], version 3.1.2) (30). In this study, BMDL_10_ was used to determine the threshold of chemical-induced biological end points, apoptosis, MMP, and ROS.

### Real-time quantitative RT–PCR

Total RNA was extracted with Trizol reagent (Invitrogen), and complementary DNA was synthesized from 1 μg of total RNA using PrimeScript RT Master Mix. Quantitative real-time PCR was performed by SYBR Green PCR Master Mix (Toyobo), and mRNA expression levels were measured by Applied Biosystems 7500 Real-Time PCR Systems. The relative expression level was calculated by 2^−ΔΔCT^ method and normalized with β-actin gene. The primers used for qRT–PCR are listed in Table S7.

### Immunoblotting and co-IP assays

For co-IP assay, cell pellets or fresh liver tissues were homogenized in ice-cold radioimmunoprecipitation assay lysis buffer (Beyotime) containing protease and phosphatase inhibitor (Roche Applied Science). The lysates were centrifuged at 12,000*g* for 20 min at 4 °C. About 3 mg of supernatant was incubated with specific antibody overnight at 4 °C and followed by the addition of protein G-Sepharose beads (GE Healthcare) for 2 h at 4 °C. The protein G beads were eluted in 2× SDS sample buffer followed by SDS-PAGE and immunoblotting. For the immunoblotting analysis of phosphorylated protein, cells were lysed directly on the plate using 2× SDS loading sample buffer. Soluble proteins (30 μl) were subjected to 3 to 8% gradient acrylamide gel for SDS-polyacrylamide gel electrophoresis before immunoblotting. The primary antibodies used in this study are listed in Table S8.

### Immunofluorescence analysis

HepG2 cells or HepG2-ADR cells (human hepatocarcinoma cells resistance to ADR) were fixed with 4% paraformaldehyde for 15 min, permeabilized with 0.2% Triton X-100 for 5 min, washed with PBS–Tween, and blocked by 3% fetal bovine serum for 1 h. Next, HepaRG cells were incubated with primary antibody against MDR1 (1:500 dilution) overnight and followed by incubation with second antibody Alexa Fluor 488-conjugated goat anti-rabbit IgG (1:1000 dilution) for 1 h. Next, the slides were counterstained with 4,6-diamidino-2-phenylindole (1 μg/ml) and examined with an LSM510 META laser scanning confocal (Leica) under a magnification 200×. Quantification was performed using ImageJ software (National Institute of Mental Health), and the results were expressed as a percent of positive staining area.

### Rh123 accumulation experiment

HepG2-ADR cells were seeded into 6-well plates at a density of 2 × 10^5^/ml and cultured overnight. About 24 h after seeding, the cells were incubated with 10 μM Rh123 for 1 h and followed by the washing with ice-cold PBS. The accumulation of Rh123 in cells was quantitatively determined by fluorescence inverted microscope with an excitation wavelength of 488 nm with 100× magnification (Leica).

### Statistical analysis

Data are shown as the mean ± SD. All statistical analyses were performed by GraphPad Prism software (GraphPad Software, Inc). Statistical differences between groups were determined by Student's *t* test (two groups) or one-way ANOVA (for more than two groups). The difference was considered statistically significant at *p* < 0.05.

## Data availability

All data are contained within the article. This article contains supporting information, including five supporting figures, eight supporting tables, and seven supporting information.

## Supporting information

This article contains supporting information.

## Conflict of interest

The authors declare that they have no conflicts of interest with the contents of this article.
