# Supplementary material for: Protein phosphatase 2A regulates cytotoxicity and drug resistance by dephosphorylating AHR and MDR1
Source: J Biol Chem. 2022 Apr 8;298(5):101918. doi: 10.1016/j.jbc.2022.101918 (PMC9118923; doi:10.1016/j.jbc.2022.101918)
Supplement: Supplemental Figures S1–S5, Tables S1–S8, Data S1–S7 [file mmc1.pdf]

Protein phosphatase 2A regulates cytotoxicity and drug resistance by dephosphorylating xenobiotic metabolism enzymes AHR and MDR1

Liping Chen[a,#], Ping Guo[a,#], Wenxue Li[b,#], Xinhang Jiang[a], Qun Zhao[c], Daochuan Li[a], Qing Wang[a], Yongmei Xiao[a], Xiumei Xing[a], Michael Aschner[d], Yiguo Jiang[e], Lihua Zhang[c,*], and Wen Chen[a,*]

Supplementary figure legends

Fig. S1 The function analysis of differentially expressed genes (DEGs) in liver tissue from HO mouse

GO analysis of differentially expressed genes (DEGs). The biological process (BP), cellular component (CC), and the molecular function (MF) were annotated and shown as red, yellow, and green colors, respectively.

Fig. S2 Activation of PP2A attenuated the decline of CYP450 gene in primary mouse hepatocytes

(**A**) Primary hepatocytes were isolated from WT or HO mice and cultured for 4, 12, 24, and 48 h, respectively. Hepatocyte morphology was examined and viewed under light microscopy (100X magnification). (**B**) The level of Aα, CYP2E1, CYP1A1, CYP1A2, CYP2A5, MDR1, and ABCG2 were determined by immunoblotting. The primary hepatocytes isolated from WT mouse were treated with FTY720 for 24 h and subjected to the examination of the mRNA (**C**) and protein expression (**D**) of Aα, CYP2E1, CYP1A1, CYP2A5, MDR1, and ABCG2. (**E**) The enzyme activity of Cyp1a1, Cyp1a2, Cyp2a5, and Cyp2e1 were measured by ELISA assay. *P<0.05, compared with the DMSO control group.

Fig. S3 PP2A was involved in chemical-induced hepatotoxicity by regulating the expression of metabolic enzymes

(**A**) Primary hepatocytes were isolated from WT or HO mice and subjected to the treatment of Bap, 5-fluorouracil, or amiodarone at indicated concentration, respectively for 24 h. The cell viability at indicated concentrations was examined by MTT assay. *, P<0.05 compared with the corresponding control cells. (**B**) The fold change in mRNA levels of Cyp1a1, Cyp1b1, Cyp1a2, Cyp2b10, Cyp3a11, Cyp2a5, Cyp2e1, Cyp3a41 in hepatocytes from WT mice treated with 20 μM BaP, 5 μM 3-methylcholane, 1 mM phenobarbital, 10 μM rifampicin, 10 μM pyrazole, 50 mM ethanol, or 5 μM amiodarone for 24 h, respectively. *P<0.05, compared with the WT mice group. (**C**) Primary hepatocytes were treated with 5-fluorouracil, cisplatin, or paclitaxel, respectively, and followed by examination of mRNA expression of Mdr1a, Abcc3, or Abcg2. (**D**) The immunoblotting analysis of PP2A Aα, MDR1, and ABCG2 in primary hepatocytes from WT and HO mice treated with 2.5 μM 5-fluorouracil for 24 h.

Fig. S4 The cytotoxicity in HepaRG cells with specific PP2A B subunit suppression

The differentiation of HepaRG cells were induced by 1.7% DMSO treatment for 14 days. (**A**) Representative image of Alb-stained HepaRG cells with 100X magnification. (**B**) The fold change in mRNA expression of Cyp3a4, Cyp1a1, and Cyp1a2 in differentiated HepaRG cells in relative to undifferentiated HepaRG cells. Schematic diagram showed the $BMDL_{10}$ of indicated cytotoxicity endpoint in indicated HepaRG cells treated with BaP **(C)** or 5-fluorouracil (**D**).

Fig. S5 PP2A was involved in regulation of the protein expression of transporters.

HepG2 cells were transfected with vectors encoding shB56δ to generate HepG2-SHB56δ cells. (**A**) The protein levels of B56δ and MDR1 were determined by immunoblotting analysis. (**B**) The immunofluorescence analysis of these cells stained with an antibody against MDR1 (green). The nuclei (blue) were stained with DAPI. Quantification of fluorescent intensity was expressed as a percentage of fields occupied (mean ± SD).

Table S1 The number of various molecular type of DEGs and up-regulated phosphoproteins

Molecular type	Transcriptomics	Phosphopepteomics
Enzyme	573	147
Transcription regulator	281	40
Transporter	163	37
Kinase	132	35
Peptidase/phosphatase	116	18

Table S2 The relative cell viability (%) of primary hepatocytes treated with chemicals/drugs, mean and SD are shown.

	WT	HO		WT	HO
Rifampicin (μM)			3-Methylcholanthrene (μM)		
0	100.0±1.0	100.0±2.0	0	100±1.2	100.0±3.0
10	97.9±0.8	97.0±0.9	10	94.1±1.6*	98.1±0.9
20	94.1±0.9*	92.6±0.7*	25	90.9±0.8*	93.8±0.8*
40	94.2±0.7*	92.6±0.7*	50	80.6±2.1*	88.5±1.4*
Phenobarbital (mM)			Pyrazole (μM)		
0	100.0±1.4	100.0±4.6	0	100.0±1.4	100.0±0.4
1	95.8±0.7	96.4±0.9	10	86.8±2.2*	93.7±1.9*
5	94.6±0.7*	95.5±0.8	20	65.5±2.7*	84.5±3.8*
10	90.3±0.6*	90.0±0.7*	40	45.2±3.9*	60.0±3.1*
Caffeine (mM)			Ethanol (mM)		
0	100.0±4.2	100.0±1.4	0	100.0±8.2	100.0±2.4
0.5	95.6±0.9	91.3±0.9*	100	44.0±3.0*	62.2±4.7*
1.0	87.7±0.9*	85.5±0.7*	200	39.1±3.5*	56.5±0.8*
2.0	83.5±0.7*	80.5±0.7*	300	35.3±0.7*	53.7±1.2*
Digoxin (μM)			Acetaminophen (μM)		
0	100.0±0.7	100.0±0.6	0	100.0±0.3	100.0±3.8
1	98.8±0.7	97.6±0.9	1	95.1±0.3	96.8±1.3
5	97.1±0.7	95.9±0.8	5	89.6±0.1*	91.5±0.4*
10	95.5±0.6	95.1±0.1	10	78.4±1.7*	85.2±1.0*
Paclitaxel (μM)			Adriamycin (μM)		
0	100.0±2.4	100.0±1.4	0	100.0±1.5	100.0±3.5
10	61.0±1.2*	66.6±2.6*	2.5	85.8±1.7*	93.3±1.1*
50	44.8±1.3*	51.5±2.3*	5.0	80.9±1.0*	88.4±0.9*
100	38.0±1.5*	42.9±1.5*	10.0	74.7±1.3*	83.5±1.3*
Cisplatin (μM)			Methotrexate (μM)		
0	100.0±2.0	100.0±1.1	0	100.0±0.7	100.0±2.3
30	55.5±1.5*	71.9±2.3*	50	86.3±1.1*	90.9±1.5*
60	49.1±2.1*	64.2±1.3*	100	80.2±1.2*	83.7±1.7*
120	40.5±0.6*	51.6±1.7*	200	73.1±1.7*	80.0±1.1*

*, were considered significantly difference compared to untreated group.

Table S3 Relative mRNA levels of metabolic enzymes induced by chemicals/drugs

	SHGFP	SHAα	SHB56α	SHB55α	SHB56δ	SHB56ε
CYP1A1	67.4	21.2*	16.4*	25.2*	23.3*	27.1*
CYP1A2	48.8	26.8*	38.6*	47.6	40.6	44.6
CYP1B1	6.7	4.3*	3.9*	6.3	4.1*	7.0
CYP2A6	2.9	2.0	1.6*	2.6	2.7	1.7*
CYP2B6	4.1	2.1*	2.2*	2.4*	4.8	4.2
CYP3A4	11.1	3.7*	1.9*	10.7	3.2*	3.5*
CYP3A5	3.7	1.4*	1.3*	3.5	3.4	1.9*
CYP2C8	3.3	1.9*	2.2*	1.6*	3.2	3.2
CYP2C9	6.6	3.4*	3.0*	2.8*	8.0	8.3
CYP2C18	13.3	3.9*	3.6*	3.4*	11.0	12.8
CYP2C19	11.1	3.7*	2.9*	2.7*	10.2	9.5
CYP2E1	3.1	1.7*	1.6*	2.8	3.2	3.1
CYP2D6	3.4	1.3*	3.3	3.5	1.3*	3.5
CYP3A7	4.9	2.0*	1.7*	2.5*	2.2*	1.1*
CYP4A11	2.3	1.2	1.1	1.2	1.4	1.2
MDR1	1.2	1.2	1.1	1.2	1.5	1.3
ABCC1	1.1	1.2	1.4	1.1	1.0	1.2
ABCC2	1.5	1.1	1.2	1.1	1.2	1.3
ABCC3	1.4	1.2	1.3	1.2	1.2	1.1
ABCG2	1.0	1.2	1.2	1.3	1.2	1.1

*, were considered significantly difference compared to SHGFP cells.

Table S4 The family subtypes CYP450 in human and mouse

Subfamily	human	mouse
CYP2A	CYP2A6	CYP2A5
CYP2B	CYP2B6	CYP2B10
CYP2C	CYP2C8	CYP2C6
	CYP2C9	CYP2C29
	CYP2C18	CYP2C40
	CYP2C19	CYP2C54
CYP2D	CYP2D6	CYP2D26
		CYP2D22
CYP2F	CYP2F1	CYP2F2
CYP3A	CYP3A4	CYP3A11
	CYP3A5	CYP3A41
	CYP3A7	CYP3A44
CYP4A	CYP4A11	CYP4A10
		CYP4A12
		CYP4A14

Table S5 The $BMDL_{10}$ (μM/mM) of various endpoints in response to chemicals/drugs in HepaRG-SHGFP cells.

Chemicals	TMRM	ROS	Apoptosis
Benzo(a)pyrene (μM)	17.75	20.53	45.51
Pyrazole (μM)	7.93	6.57	8.92
Phenobarbital (mM)	4.97	5.84	2.85
Rifampicin (μM)	48.47	56.40	77.46
Acetaminophen (μM)	0.49	0.76	0.88
Amiodarone (μM)	1.13	0.93	1.21
5-Fluorouracil (μM)	0.40	0.52	2.86
Methotrexate (μM)	5.98	3.48	7.80
Cisplatin (μM)	2.71	8.34	9.07

Table S6 The $BMDL_{10}$ (μM/mM) of sensitive endpoints

Chemicals	Endpoints	SHGFP	SHAα	SHB56α	SHB55α	SHB56δ	SHB56ε
Benzo(a)pyrene (μM)	TMRM	17.75	26.55*	26.74*	18.95	19.27	16.59
Pyrazole (μM)	ROS	6.57	10.68*	10.90*	6.18	6.35	5.97
Phenobarbital (mM)	Apotposis	2.85	2.75	2.79	2.82	2.53	2.48
Rifampicin (μM)	TMRM	48.47	48.32	48.19	48.19	48.55	48.36
Acetaminophen (μM)	TMRM	0.49	0.77*	0.67*	0.37*	0.52	0.47
Amiodarone (μM)	ROS	0.93	0.77*	0.74*	0.78*	0.87	0.74*
5-Fluorouracil (μM)	TMRM	0.40	1.42*	0.53	0.32	1.47*	0.36
Methotrexate (μM)	ROS	3.48	7.13*	2.48	3.96	6.09*	3.12
Cisplatin (μM)	TMRM	2.71	3.58	2.13	2.93	3.77	2.65

*, were considered significantly difference compared to SHGFP cells.

Table S7 Primers for q-RT-PCR assay

gene	Primer sequences (forward and reverse)
Cyp1a1	F:GTGAGCAAGGAGGCTAACTATC
	R:GGCTACTGACACGACCAAATA
Cyp1a2	F:GTCTTCCTCTTCTTAGCCATCC
	R:GCTTCATGGTCAACCCATAGT
Cyp1b1	F:TGGCCCTTTCCTCCTATCT
	R:ACTGACACAACCTGCGTATC
Cyp2a5	F:GAGGAGATTGATCGGGTGATTG
	R:CATGGATTACAGCCTCCGTATAG
Cyp2a22	F:GGCACTGATGTGTTCCCTATAA
	R:TCTTCAACTGTCCCTTGTCATC
Cyp3c38	GCTCTGCTTCATTCCTCTCTAAA
	CCAGGATGGAAGATGCCTTATC
Cyp2c37	F:GCTTGAACACCTGGCAATAAC
	R:GGGTACTTCAAGAGGAGTAGGA
Cyp2c40	F:CACAGCTAAAGTCCAGGAAGAG
	R:GCACCATGGCATTTGTGTAAG
Cyp2c50	F:CTGCCAATCCTTCACCAATTTATC
	R:CTTCCTTCACTGCCTCATATCC
Cyp2d9	F:CAGAAGTCCTTCATCGCCATAC
	R:CCAGGAAGGCATCAGTCAAA
Cyp2e1	F:GAAGTCTCTGGTTGACCCTAAG
	R:AGGTCTCATGAACGAGGAATG
Cyp3a41a	F:ACCACCAGTAGCACACTTTC
	R:CCTTATTGGGCAGAGTCTCATC
Cyp3a44	F:CACACTTTCCTTCACCTTGTATTG
	R:CAGGTGGGAGTTGCCTTATT
Cyp3a57	F:GTACCCAGTTGTTAGCAGGATAG
	R:GTCCCGATGAAGAGGATAGATTG
Cyp4a10	F:CCCTGATGGACGCTCTTTAC
	R:GGGTCAAACACCTCTGGATT
Fmo1	F:GATGACCTCCTGACCTCTATCA
	R:GGTACATGGGCCAAAGAAGA
Fmo2	F:GATGAACTCGCCTTGGAGATAG
	R:GCGGTACTGGTAGGAATTACAG
Fmo3	F:CACCACCATCCAGACAGATTAC
	R:CCTTGAGAAACAGCCATAGGAG
Fmo4	F:ATGGGATGGAGCCAGAAATG
	R:AGTAATGTGAGAGGGAGGTAGG
Fmo5	F:GGCTGGCATTACGGTTACT
	R:CGATCTTCCGTGGTGAGAATAG

Gstm1	F:CTCACGCTTCCTAGAATTACCC
	R:CAGGCTGGCACTCAAGTATT
Gstm6	F:GCCCAAGTCTCTGGGTTATT
	R:CCATGGCGTATCTCTTCTCTTC
Gstm7	F:AGGGTGGGCTGTAGGATAAA
	R:CTGGAACAGGATGGAAGGAAAG
Gstt2	F:GTACTTTCGGAGTGCTCCTATG
	R:GCAGAACCAGGACCATTCTATC
Ugcg	F:TGGTTCTAGGAAGTGGGATTTG
	R:CTGCAGAGCAGTCCTGTATATG
Uggt1	F:TGGTGGTGATGAAGGACATTAG
	R:TCTCTTCCACTTCCGCTCT
Ugt1a10	F:CCTCTTTAGCCCAGTGTCTATTT
	R:GACAGTTCATCCCACCAACA
Ugt1a2	F:GCTGTGTTCTGGGTGGAATA
	R:CATCCAAGGAGTGGTACTGATAC
Ugt1a5	F:CATGAGGGATGTTGTGAGAGAG
	R:GTCTTGTATAGGGAACGGCATAG
Ugt1a6a	F:TGAAGGAGATACAGGGATAGAGG
	R:TGCTTTGGGACCTGTGTAAG
Mdr1a	F:GCCTACTATTACACCGGGATTG
	R:CTTCTGCCTGATCTTGTGTATCT
Abca5	F:GACTGAAGGTCCCTATCCTCTAA
	R:CTGGAATGACTTGGTCGAGATAC
Abcb8	F:TGGAACCCTCGGTAGGATAA
	R:CCGGATTCCGAACCGAAATA
Abcc10	F:CCGTGTGTGTCTTCTCATTCT
	R:CCATCTTCAGCTACCTCTGTTT
Abcg2	F:GATGAACTCCAGAGCCGTTAG
	R:CGGACTAGAAACCCACTCTTTAC
Slc19a1	F:TCGCCTACTCCTCCTACATATT
	R:CCCAACACAGAGCTGATGAA
Slc26a4	F:GCAAGCACACTCACTCTTCTA
	R:GTCTGCCAAGTACCTCACTATG
Slc27a3	F:CAAGGCAGAGTGTGACCATAA
	R:CATGCCATACGTCTCCAGTATC
Slc30a6	F:GAGATACACACGGGAAGATTGT
	R:CTAGCAGCTTCAGAGACGTAAG
Slc35a4	F:GACTCATCTCAGGGAACATTGG
	R:CCTAAGTGCTTCAGAGGGAAAG
Slc35d2	F:GTCAGGCATGGAGCATATCA
	R:AACCTGATGCTTGCCATTTAAG
Slc43a1	F:CAGCTGAGAACAGGACCAATAC
	R:GGAAGGAGCCAATGGTGAAA

Slc45a2	F:AAGCTGATCTGGGCCATAAG
	R:GGTGGGAGCAGACATCAAATA
Slc47a1	F:GTTCTGGGCCCTCTAACATAAG
	R:CCCTCTCTCTGTCTCTCTCAAA
Slc6a12	F:CTTGTCACCTCTCAGTCCTTTC
	R:CTTTGCCTGTCTGGTTCTTAGT

Table S8 Antibodies used in this study

Antibody	Brand	Catalogue Number
phosphoserine/threonine	BD Biosciences	612549
PP2A Cα	BD Biosciences	41006
PP2A C (ID6)	Upstate Biotechnology	05-421
PP2A B55α	Dr. W.C. Hahn's lab	/
PP2A B56ε	Dr. W.C. Hahn's lab	/
CYP1A1	Bioworld	BS6575
CYP1A2	Cell Signaling Technology	14719
MDR1	Cell Signaling Technology	13978/13342
ABCG2	Cell Signaling Technology	42078
PP2A B56δ	Dr. W.C. Hahn's lab	
PP2A Aα	Cell Signaling Technology	2039
CYP2E1	Proteintech Group	19937-1-AP
CYP2A6/5	Proteintech Group	21721-1-AP
β-actin	Proteintech Group	66009-1-Ig
PP2A B56α	Novus Biologicals	NB100-41412
AHR (phospho-Ser36)	GeneTex	GTX52324
AhR	Santa Cruz Biotechnology	sc-133088

Supplemtentary data

Data S1 Differentially expressed transcripts and genes in HO mouse.

Data S2 Up-regulated phosphoproteins and phosphorylation sites in HO mouse.

Data S3 The perturbed canonical pathways attributable to PP2A inactivation in two omics datasets.

Data S4 The altered metabolic enzyme genes in HO mouse liver.

Data S5 The validated 60 metabolic DEGs by proteomics analysis.

Data S6 The metabolic enzymes targeted by transcriptional regulators.

Data S7 The regulatory network predicted by IPA analysis between PP2A subunits and metabolic enzymes.

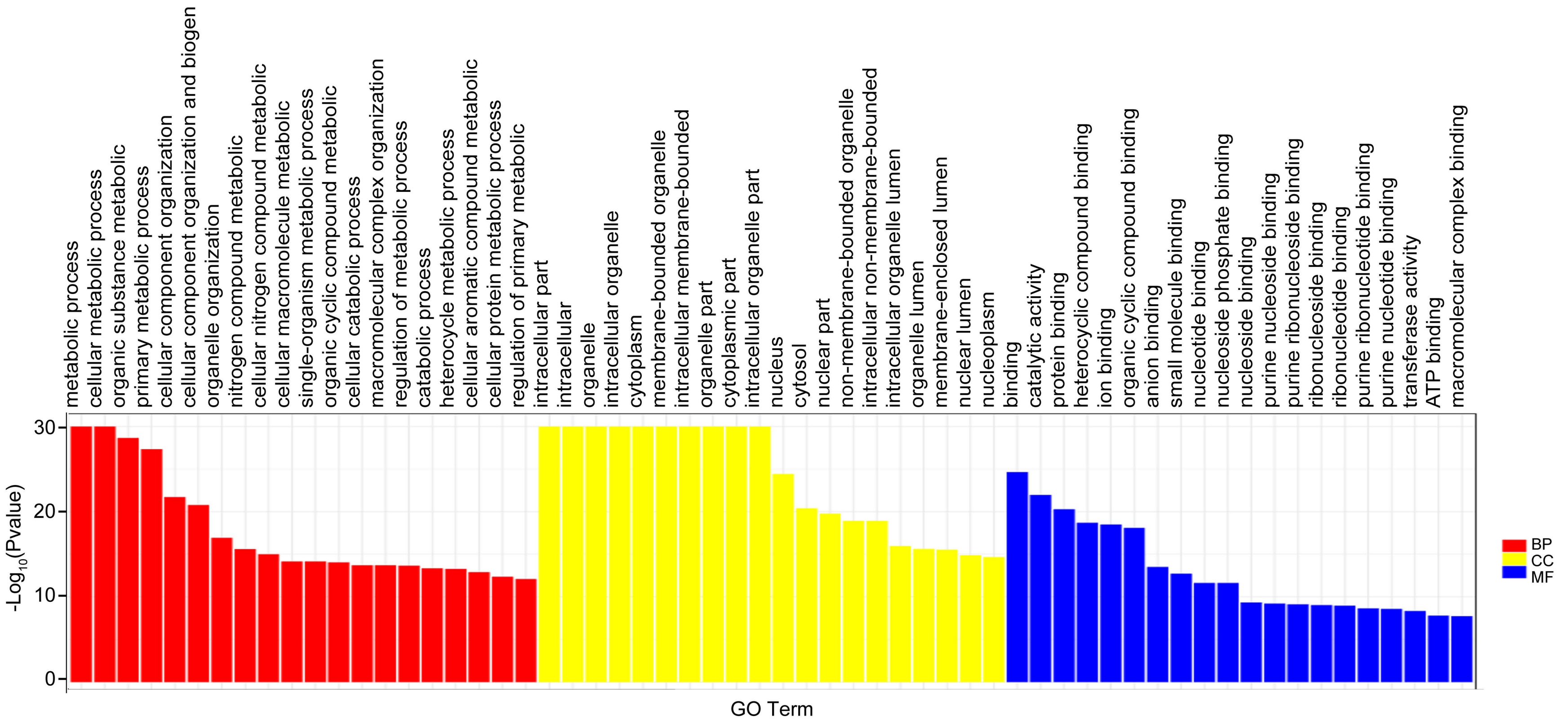

metabolic process
cellular metabolic process
organic substance metabolic
primary metabolic process
cellular component organization
cellular component organization and biogen
organelle organization
nitrogen compound metabolic
cellular nitrogen compound metabolic
cellular macromolecule metabolic
single-organism metabolic process
organic cyclic compound metabolic
cellular catabolic process
macromolecular complex organization
regulation of metabolic process
catabolic process
heterocycle metabolic process
cellular aromatic compound metabolic
cellular protein metabolic process
regulation of primary metabolic
intracellular part
intracellular
organelle
intracellular organelle
cytoplasm
membrane-bounded organelle
intracellular membrane-bounded
organelle part
cytoplasmic part
intracellular organelle part
nucleus
cytosol
nuclear part
non-membrane-bounded organelle
intracellular non-membrane-bounded
intracellular organelle lumen
organelle lumen
membrane-enclosed lumen
nuclear lumen
nucleoplasm
binding
catalytic activity
protein binding
heterocyclic compound binding
ion binding
organic cyclic compound binding
anion binding
small molecule binding
nucleotide binding
nucleoside phosphate binding
nucleoside binding
purine nucleoside binding
purine ribonucleoside binding
ribonucleoside binding
ribonucleotide binding
purine ribonucleotide binding
purine nucleotide binding
transferase activity
ATP binding
macromolecular complex binding
30
20
10
0
-Log10(Pvalue)
GO Term
BP
CC
MF

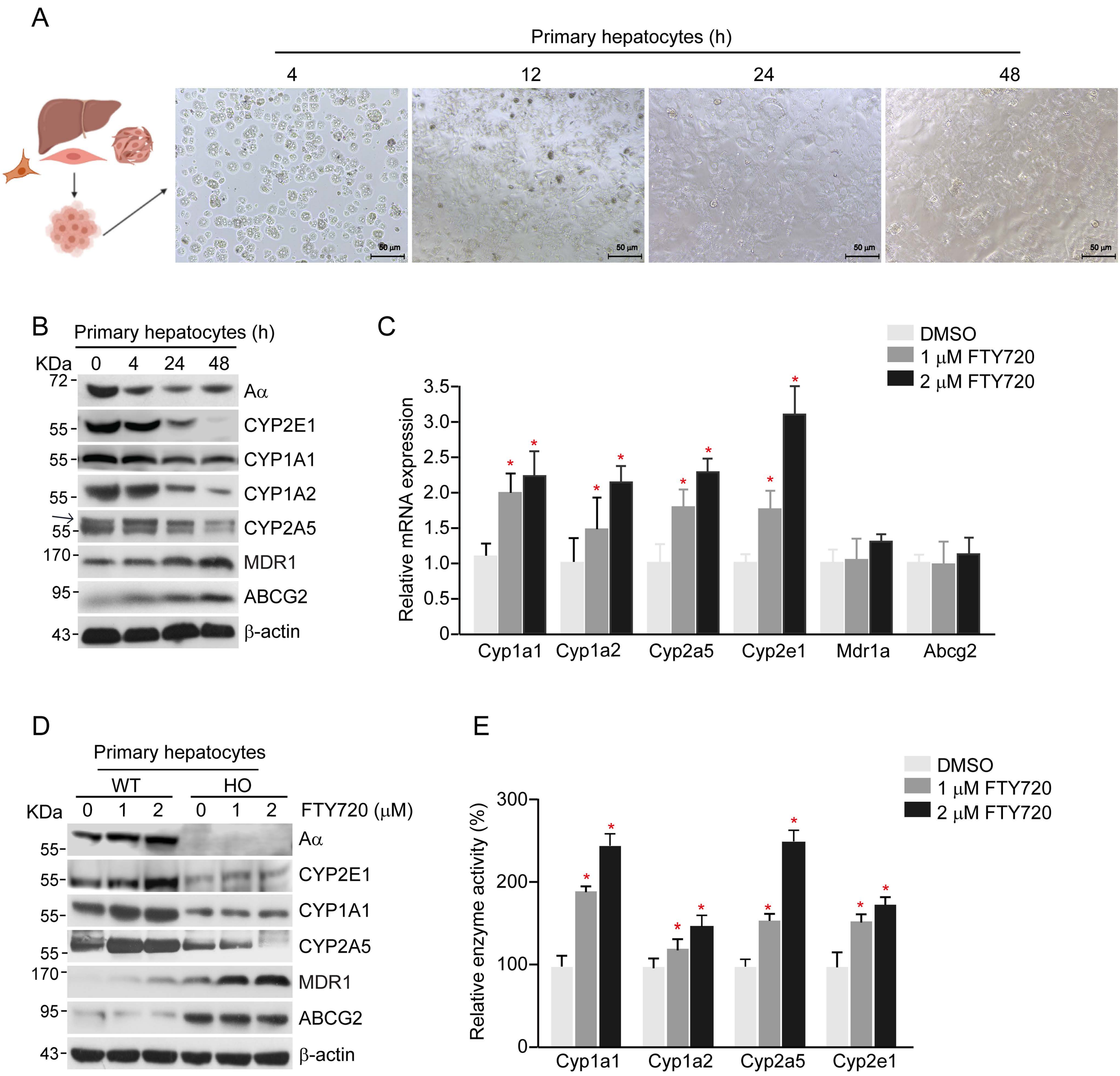
A
Primary hepatocytes (h)
4
12
24
48
50 μm
B
Primary hepatocytes (h)
KDa
0 4 24 48
72
55
55
55
55
170
95
43
Aα
CYP2E1
CYP1A1
CYP1A2
CYP2A5
MDR1
ABCG2
β-actin
C
DMSO
1 μM FTY720
2 μM FTY720
Relative mRNA expression
Cyp1a1
Cyp1a2
Cyp2a5
Cyp2e1
Mdr1a
Abcg2
D
Primary hepatocytes
WT
HO
FTY720 (μM)
E
Relative enzyme activity (%)

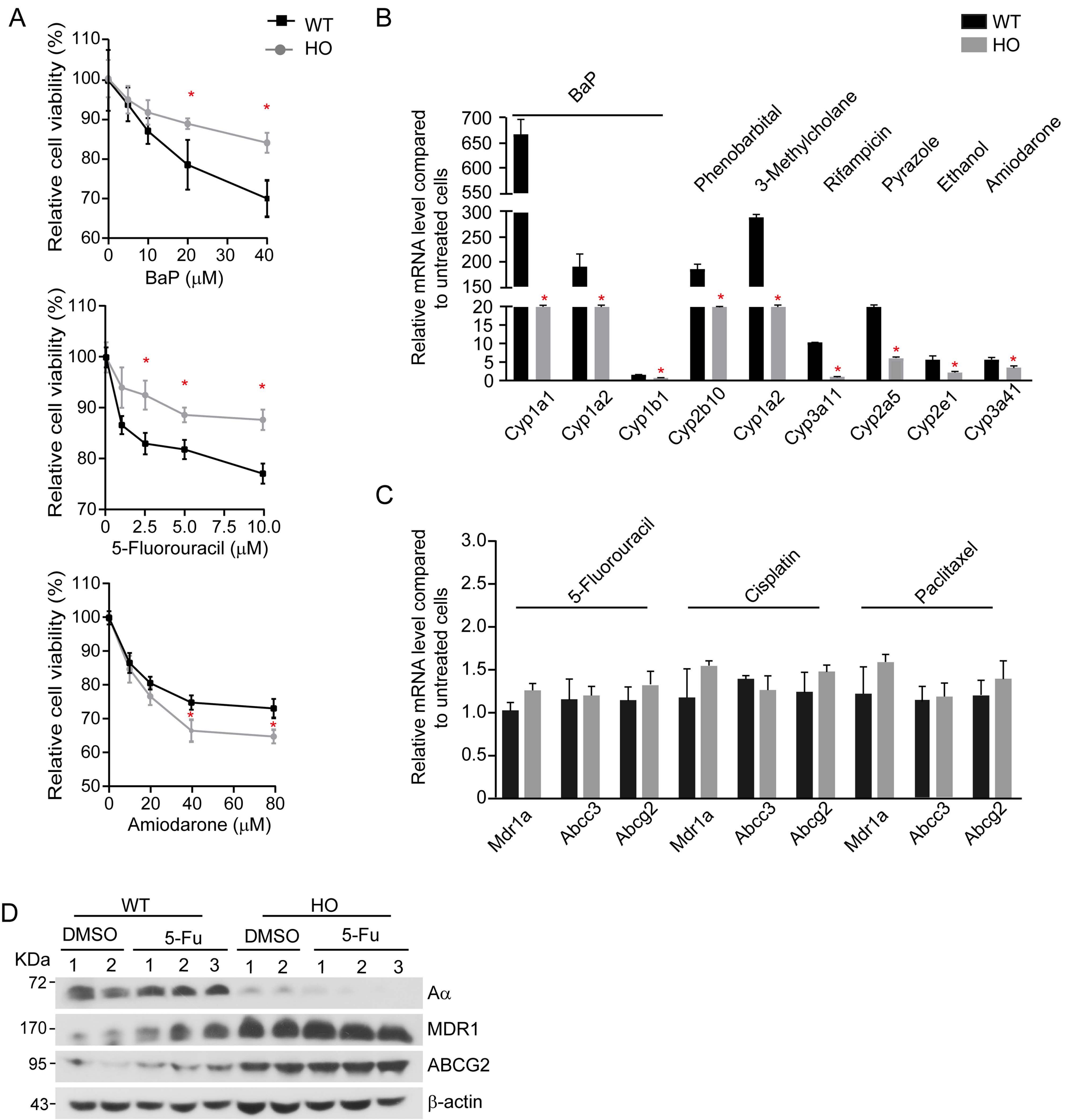
A
WT
HO
Relative cell viability (%)
BaP (μM)
5-Fluorouracil (μM)
Amiodarone (μM)
B
BaP
Phenobarbital
3-Methylcholane
Rifampicin
Pyrazole
Ethanol
Amiodarone
Relative mRNA level compared to untreated cells
Cyp1a1
Cyp1a2
Cyp1b1
Cyp2b10
Cyp1a2
Cyp3a11
Cyp2a5
Cyp2e1
Cyp3a41
C
5-Fluorouracil
Cisplatin
Paclitaxel
Relative mRNA level compared to untreated cells
Mdr1a
Abcc3
Abcg2
D
WT
HO
DMSO
5-Fu
KDa
72
170
95
43
Aα
MDR1
ABCG2
β-actin

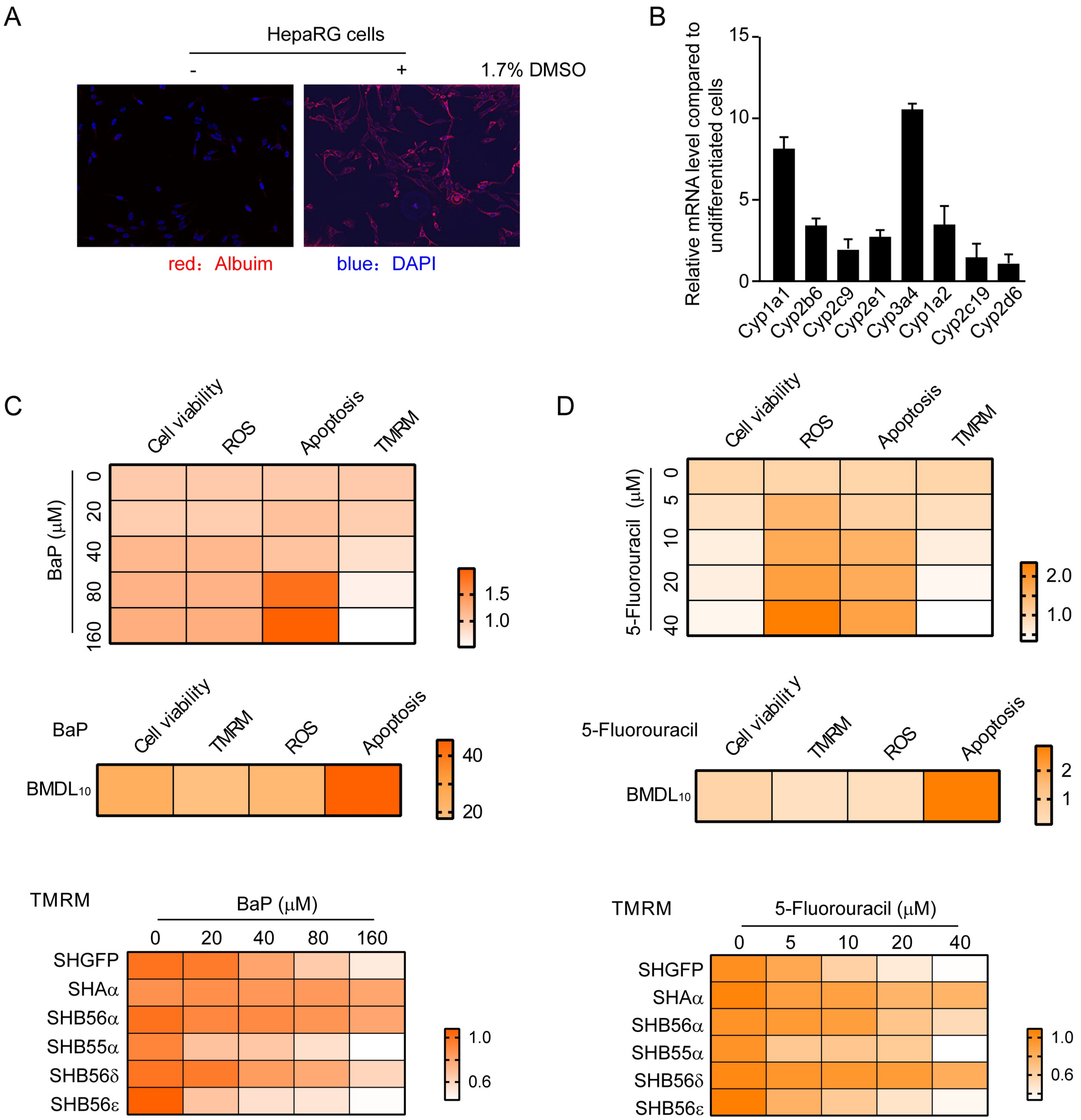
A
HepaRG cells
-
+
1.7% DMSO
red：Albuim
blue：DAPI
B
Relative mRNA level compared to undifferentiated cells
15
10
5
0
Cyp1a1
Cyp2b6
Cyp2c9
Cyp2e1
Cyp3a4
Cyp1a2
Cyp2c19
Cyp2d6
C
Cell viability
ROS
Apoptosis
TMRM
BaP (μM)
0
20
40
80
160
1.5
1.0
D
5-Fluorouracil (μM)
0
5
10
20
40
2.0
1.0
BaP
TMRM
BMDL10
40
20
5-Fluorouracil
2
1
TMRM
BaP (μM)
SHGFP
SHAα
SHB56α
SHB55α
SHB56δ
SHB56ε
1.0
0.6
5-Fluorouracil (μM)

A

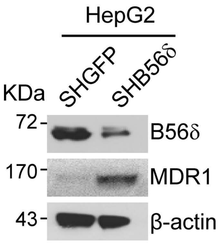
HepG2
SHGFP
SHB56δ
KDa
72
170
43
B56δ
MDR1
β-actin

B

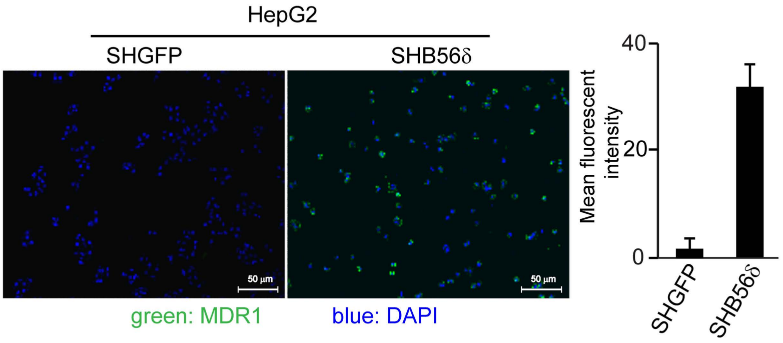
HepG2
SHGFP
SHB56δ
50 μm
50 μm
green: MDR1
blue: DAPI
Mean fluorescent intensity
40
20
0
SHGFP
SHB56δ
